# High expression of *HMGA2* independently predicts poor clinical outcomes in acute myeloid leukemia

**DOI:** 10.1038/s41408-018-0103-6

**Published:** 2018-07-19

**Authors:** Miriam Marquis, Cyrielle Beaubois, Vincent-Philippe Lavallée, Michal Abrahamowicz, Coraline Danieli, Sébastien Lemieux, Imran Ahmad, Andrew Wei, Stephen B. Ting, Shaun Fleming, Anthony Schwarer, David Grimwade, William Grey, Robert K. Hills, Paresh Vyas, Nigel Russell, Guy Sauvageau, Josée Hébert

**Affiliations:** 1 The Quebec Leukemia Cell Bank, Research Centre, Maisonneuve-Rosemont Hospital, Montréal, Canada; 2 The Leucegene project at Institute for Research in Immunology and Cancer, Université de Montréal, Montréal, Canada; 3 Division of Hematology-Oncology, Maisonneuve-Rosemont Hospital, Montréal, Canada; 4 Epidemiology and Biostatistics Department, McGill University, Montréal, Canada; 5 Department of Computer Science and Operations Research, Université de Montréal, Montréal, Canada; 6 Department of Medicine, Faculty of Medicine, Université de Montréal, Montréal, Canada; 7 Department of Haematology, Alfred Hospital, Melbourne, Australia; 8 Australian Centre for Blood Diseases, Monash University, Melbourne, Australia; 9 Faculty of Medicine, Nursing and Health Sciences, Monash University, Melbourne, Australia; 10 Department of Haematology, Eastern Health, Box Hill Hospital, Melbourne, Australia; 11 Cancer Genetics Laboratory, Department of Medical and Molecular Genetics, King’s College London, London, UK; 12 UK National Cancer Research Institute (NCRI) Haematological Oncology Clinical Studies Group, Cardiff, UK; 13 Centre for Trials Research, Cardiff University School of Medicine, Cardiff, UK; 14 MRC Molecular Haematology Unit, Weatherall Institute of Molecular Medicine and Department of Haematology, University of Oxford and Oxford University Hospitals NHS Trust, Oxford, UK; 15 NIHR Oxford Biomedical Research Centre, Oxford, UK; 16 Centre for Clinical Haematology, Nottingham University Hospital (City Hospital Campus), Nottingham, UK

## Abstract

In acute myeloid leukemia (AML), risk stratification based on cytogenetics and mutation profiling is essential but remains insufficient to select the optimal therapy. Accurate biomarkers are needed to improve prognostic assessment. We analyzed RNA sequencing and survival data of 430 AML patients and identified *HMGA2* as a novel prognostic marker. We validated a quantitative PCR test to study the association of *HMGA2* expression with clinical outcomes in 358 AML samples. In this training cohort, *HMGA2* was highly expressed in 22.3% of AML, mostly in patients with intermediate or adverse cytogenetics. High expression levels of *HMGA2* (*H* *+* ) were associated with a lower frequency of complete remission (58.8% vs 83.4%, *P* *<* 0.001), worse 3-year overall survival (OS, 13.2% vs 43.5%, *P* *<* 0.001) and relapse-free survival (RFS, 10.8% vs 44.2%, *P* *<* 0.001). A positive HMGA2 test also identified a subgroup of patients unresponsive to standard treatments. Multivariable analyses showed that *H* *+* was independently associated with significantly worse OS and RFS, including in the intermediate cytogenetic risk category. These associations were confirmed in a validation cohort of 260 patient samples from the UK NCRI AML17 trial. The HMGA2 test could be implemented in clinical trials developing novel therapeutic strategies for high-risk AML.

## Introduction

In adult acute myeloid leukemia (AML), clinical outcome is predicted by age, cytogenetics and specific gene mutations.^1–5^ In the recent European LeukemiaNet (ELN) guidelines for AML genetic testing, screening for mutations in *NPM1*, *CEBPA*, *RUNX1, FLT3*, *TP53*, and *ASXL1* genes in addition to chromosomal anomalies is recommended.^1^ It is now well accepted that the genetic and cytogenetic risk stratification guides AML consolidation therapy: patients in a favorable risk category are treated with conventional consolidation chemotherapy, whereas adverse-risk patients are usually referred for allogeneic hematopoietic stem cell transplantation (allo-HSCT), a procedure carrying an inherent mortality rate surpassing 15%.^6^ However, the ideal consolidation therapy remains unclear for up to 40% of AML patients classified in the intermediate-risk category, hence the need to improve prognostic assessment in this patient subgroup.^1^ Likewise, identification of possible long-term survivors in the adverse-risk group represents another clinical challenge.

Gene expression signatures, mostly derived from microarray studies, have been evaluated as a means to further improve AML risk stratification.^7–14^ Although several markers have been identified, they have not been widely adopted because of technical challenges in implementing large gene signatures in clinical settings. Global RNA-sequencing technologies, which are more accurate in estimating gene expression levels than microarray studies,^15^ have now been applied to a few large AML cohorts including that of The Cancer Genome Atlas (TCGA, *n* = 179)^16^ and Leucegene (*n* = 430).^17–21^ These data sets provide new opportunities to determine whether candidate gene expression levels can complement currently accepted prognostic tests.

In this study, we have explored the Leucegene data set using bioinformatic tools to identify genes with bimodal expression patterns that correlate with patient survival. The two best candidate genes, High Mobility Group AT-Hook 2 (*HMGA2*) and Pro-Apoptotic *WT1* Regulator (*PAWR*) were evaluated in the training cohort but only *HMGA2* was validated in the independent cohort. We present the development and inter-laboratory validation of a RT-qPCR HMGA2 clinical test and demonstrate its utility to refine AML risk stratification.

## Patients, materials, and methods

### Study design, patients, and AML sample characteristics

**Fig. 1:**
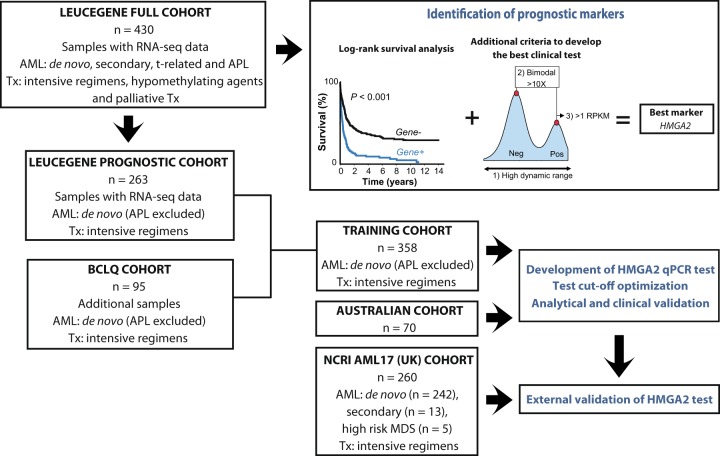
Flow diagram of the study and discovery approach for identification of *HMGA2*. The *HMGA2* prognostic marker was identified from the RNA-sequenced samples of the Leucegene full cohort (*n* = 430). Criteria for marker selection were: best log-rank *P* values to discriminate between poor vs good survivors based on the 75th percentile of expression (in RPKM values) for each gene, high dynamic range, bimodal distribution of gene expression values, and gene expression values above one RPKM. Development, analytical, and clinical validation of the HMGA2 RT-qPCR test were performed in the training cohort (*n* = 358). The Australian cohort (*n* = 70) was used to validate the RT-qPCR expression values. The HMGA2 test was externally validated in the NCRI AML17 cohort (*n* = 260). AML, acute myeloid leukemia; APL, acute promyelocytic leukemia; MDS, myelodysplastic syndromes; RPKM, reads per kilobase per million mapped reads; Tx, treatment

This study is part of the Leucegene project and was approved by the Research Ethics Boards of Université de Montréal and Maisonneuve-Rosemont Hospital. Diagnostic AML samples and clinical data were collected with informed consent from patients between 2002 and 2014 at nine hospitals participating in the Banque de cellules leucémiques du Québec program (BCLQ, bclq.org). The Leucegene full cohort of 430 RNA-sequenced samples (Fig. 1) was used for the discovery of new candidate prognostic markers. RNA-sequencing data are available separately,^17–21^ #GSE49642, #GSE52656, #GSE62190, #GSE66917, #GSE67039. The training cohort includes 263 *de novo* AML patients treated with intensive regimens sequenced in the Leucegene project and 95 additional BCLQ specimens similarly selected, which were not sequenced (Fig. 1). The median follow-up was 6.0 years. Alive patients were censored at their last follow-up (May to August 2015). Four additional patients were censored owing to loss to follow-up. Definitions of complete remission (CR), overall survival (OS), relapse-free survival (RFS) and cumulative incidence of relapse (CIR) followed ELN recommendations.^1^ Description of clinical characteristics and treatment protocols are provided in the Supplementary Information (Supplementary Figures S1–S2; Supplementary Tables S1–S4). AML samples (*n* = 70) from Australia were used to confirm the distribution of *HMGA2* expression values (Fig. 1 and Supplementary Table S5). This study was approved by the Human Research Ethics Committees of the Alfred and the Box Hill Hospitals in Melbourne. The external validation cohort included 263 AML samples from intensively treated patients enrolled in the UK NCRI AML17 trial (ISRCTN55675535) approved by Wales Research Ethics Committee 3 (Fig. 1 and Table 1). Patients with intermediate- and adverse-risk cytogenetics were selected for external validation because the HMGA2 test appears useful in these risk categories. *HMGA2* expression values were not available for 3 out of 263 samples.

**Table 1:** Association of *HMGA2* expression levels with clinical and genetic characteristics

	Training cohort					Validation cohort			
Characteristics	Total (*n* = 358)	*HMGA2* low (*n* = 278)	*HMGA2* high (*n* = 80)	*P*		Total (*n* = 260)	*HMGA2* low (*n* = 160)	*HMGA2* high (*n* = 100)	*P*
Age at diagnosis, years									
Median		53	58			51.5	50	53	0.09
Range		17–78	21–74			0–71	7–69	0–71	
Age, *n*									
0–16 years	0	0	0			12	7	5	
17–59 years	235	193	42	0.007		184	118	66	
≥ 60 years	123	85	38			64	35	29	
Male sex, *n* (%)	194	149 (53.6)	45 (56.3)	0.704		140	80 (50.0)	60 (60.0)	0.4
WBC (× 10^9^/l), *n* (%)									
<50	230	166 (59.7)	64 (80.0)	0.001		195	107 (66.9)	88 (88.0)	<0.001
50–99	76	69 (24.8)	7 (8.8)			39	33 (20.6)	6 (6.0)	
>100	48	41 (14.7)	7 (8.8)			26	20 (12.5)	6 (6.0)	
Not available	4	2 (0.8)	2 (2.4)			0	0 (0.0)	0 (0.0)	
AML history, *n* (%)									
*De novo*	358	278 (100.0)	80 (100.0)			242	152 (95.0)	90 (90.0)	0.16
Secondary	0	0 (0.0)	0 (0.0)			13	6 (3.7)	7 (7.0)	
High-risk MDS	0	0 (0.0)	0 (0.0)			5	2 (1.3)	3 (3.0)	
CR, *n* (%)	279	232 (83.4)	47 (58.8)	<0.001		207	137 (85.6)	70 (70)	0.002
HSCT, *n* (%)									
CR1	66	54 (19.4)	12 (15.0)			68	41 (25.6)	27 (27.0)	
CR2	32	27 (9.7)	5 (6.3)			25	18 (11.2)	7 (7.0)	
Others	2	0 (0.0)	2 (2.5)			13	8 (5.0)	5 (5.0)	
Cytogenetic risk, *n* (%)									
Favorable	54	51 (18.3)	3 (3.8)	<0.001		0	0 (0.0)	0 (0.0)	<0.001
Intermediate	232	191 (68.7)	41 (51.3)			214	142 (88.8)	72 (72.0)	
Adverse	68	33 (11.9)	35 (43.8)			35	11 (6.9)	24 (24.0)	
Undetermined	4	3 (1.1)	1 (1.3)			11	7 (4.4)	4 (4.0)	
2017 ELN genetic risk, *n* (%)									
Favorable	185	180 (64.7)	5 (6.3)	<0.001			—	—	
Intermediate^a^	84	54 (19.4)	30 (37.5)				—	—	
Adverse	87	42 (15.1)	45 (56.3)				—	—	
Undetermined	2	2 (0.7)	0 (0.0)				—	—	
NCRI AML17 Risk group^b^, *n* (%)									
Known high risk		—	—			101	53 (33.1)	48 (48.0)	0.05
Not high risk		—	—			157	105 (65.6)	52 (52.0)	
Not calculable		—	—			2	2 (1.3)	0 (0.0)	
WHO Performance Status									
0		—	—			160	104 (65.0)	56 (56.0)	0.4
1		—	—			84	48 (30.0)	36 (36.0)	
2		—	—			8	5 (3.1)	3 (3.0)	
3		—	—			3	2 (1.3)	1 (1.0)	
Not completed		—	—			5	1 (0.6)	4 (4.0)	
Mutations in the training cohort and the validation cohort, *n* (%)									
Intermediate cytogenetics	232	191	41		Total^c^	256	157	99	
*FLT3*-ITD	90	81 (42.4)	9 (22.0)	0.021		66	54 (34.4)	12 (12.1)	<0.001
*NPM1*	128	126 (66.0)	2 (4.9)	<0.001		113	91 (58.0)	22 (22.2)	<0.001
*NPM1−* and *FLT3-*ITD*−*	79	49 (25.7)	30 (73.2)			122	51 (32.5)	71 (71.7)	
*NPM1*− and *FLT3-*ITD− and bi*CEBPA−*	69	39 (20.4)	30 (73.2)				—	—	
*NPM1+* and *FLT3-*ITD*−*	63	61 (31.9)	2 (4.9)			69	53 (33.8)	16 (16.2)	
*NPM1*− and *FLT3-*ITD*+*	25	16 (8.4)	9 (22.0)			44	38 (24.2)	6 (6.1)	
*NPM1*+ and *FLT3*-ITD+	65	65 (34.0)	0 (0.0)			21	15 (9.6)	6 (6.1)	
Adverse-risk mutations in the sequenced cohort (excluding patients with favorable cytogenetics), *n* (%)									
Total	219	174	45						
*TP53*	19	3 (1.7)	16 (35.6)				—	—	
*RUNX1* only	14	7 (4.0)	7 (15.6)				—	—	
*ASXL1* only	5	4 (2.3)	1 (2.2)				—	—	
*RUNX1* and *ASXL1*	6	3 (1.7)	3 (6.7)				—	—	

— not available, *CR* complete remission, *HMGA2* low expression level of *HMGA2* < 1100 NCN, *HMGA2* high expression level of *HMGA2* ≥ 1100 NCN, *HSCT* allogeneic hematopoietic stem cell transplantation, *MDS* myelodysplastic syndromes, *WBC* white blood cells

^a^Thirty-seven patients with intermediate-risk cytogenetics and absence of *NPM1*, *FLT3*-ITD, and biallelic *CEBPA* (bi*CEBPA*) mutations were not sequenced

^b^The NCRI high-risk category is defined in the statistical methods section in the Supplementary Information

^c^In the validation cohort, *NPM1* and *FLT3*-ITD mutation status was not available for four patients

### Cytogenetics, mutation analysis, and RNA sequencing

Cytogenetic risk was categorized according to ELN recommendations.^1^ Methods for leukemia cell cryopreservation and for *NPM1*, *FLT3*-ITD, and *CEBPA* mutation testing are described in the Supplementary Information. The workflow for RNA-sequencing and mutation analysis has been described previously.^20^

### Quantitative PCR experiments

A RT-qPCR assay to evaluate *HMGA2* expression was developed. Detailed methods including complementary DNA synthesis, primer, and probe sequences, PCR, construction of plasmid standard curves and results of analytical validation are outlined in the Supplementary Information (Methods section, Supplementary Tables S6 and S7). Normalized copy numbers (NCN) of *HMGA2* were generated following Europe Against Cancer program recommendations.^22^

### Statistical methods

Receiver operating characteristic (ROC) curves and the Youden index were used to identify a threshold between low and high *HMGA2* expression values.^23,24^ Fisher’s exact test was used to test bivariate unadjusted associations between the marker, dichotomized as above (*H* *+* ) vs. below (*H* *−*) the threshold, and categorical variables. Probabilities of OS were estimated with Kaplan–Meier curves and compared using the log-rank test. CIR curves were estimated using competing risks analyses to account for mortality and compared with Gray’s test.^25^ OS was measured from the date of AML diagnosis and RFS and CIR were measured from the date of achievement of a remission. For studies in the subgroup of younger transplanted patients, time 0 was defined as the date of transplantation. Main analyses relied on multivariable regression methods to estimate the associations of the dichotomized marker with each of the clinically relevant outcomes. Multivariable models were adjusted for the following set of established prognostic variables: age, white blood cell (WBC) counts, HSCT as a time-dependent variable (except for CR prediction), cytogenetic risk and *NPM1* and *FLT3*-ITD mutation status. *TP53, RUNX1*, and *ASXL1* mutations were added as variables for models in the sequenced cohort and biallelic *CEBPA*, *RUNX1*, and *ASXL1* mutations for models in the intermediate cytogenetic risk subgroup. The effect of age was modeled using the linear and quadratic terms, to account for its significantly non-linear relationships with most of the outcomes (Supplementary Information, Statistical Methods section). The ability of *HMGA2* to enhance CR prediction was assessed with multivariable logistic regression and its independent association with the time to relapse and/or death was estimated by multivariable Cox proportional hazards regression. Flexible time-dependent model was used to test the proportional hazards assumption^26^ (Supplementary Information, Statistical Methods section). The Lunn-McNeil competing risks extension of the Cox model^27,28^ estimated associations of the marker with the hazards of either relapse or death. Statistical significance of the associations was tested using multivariable model-based Wald tests and their strength quantified by the adjusted hazard ratio (HR) or for CR, odds ratio (OR), with 95% confidence intervals. All *P* values were two-sided and considered statistically significant if *P* < 0.05. The analyses were performed with R (v3.2.2) and EZR (v3.1) softwares. Statistical methods for the NCRI AML17 cohort are described in the Supplementary Information (Statistical Methods section).

This manuscript complies with the REMARK guidelines^29^ (Supplementary Table S8).

## Results

### Identification of *HMGA2* as a new prognostic marker in AML

We first investigated all annotated genes in the Leucegene full cohort (*n* = 430) for their potential to discriminate between patients with good vs. poor survival by analyzing survival based on the 75th percentile of expression values (Fig. 1). The best candidate prognostic markers were also selected for features that would ease their usage as clinical tests: (1) high dynamic range of expression; (2) evidence for bimodal distribution illustrative of two distinct subgroups with more than tenfold difference in reads per kilobase per million mapped reads (RPKM) values between low and high expressors, and (3) peak expression in high expressors above one RPKM. *HMGA2* and *PAWR* were identified for test development and validation but only *HMGA2* was validated in the independent NCRI AML17 validation cohort and is reported herein. Analyses of *PAWR* in the validation cohort are provided in Supplementary Figure S3 and Supplementary Table S9.

Notably, based on the 75th percentile of *HMGA2* expression values, most genetic anomalies associated with poor survival were highly prevalent in the *HMGA2* positive subgroup including samples with complex karyotype, *TP53* mutations or other adverse-risk mutations such as *RUNX1*, *ASXL1*, *SRSF2*, and *MLL* (Supplementary Figure S4).

### Development and validation of the HMGA2 RT-qPCR test

**Fig. 2:**
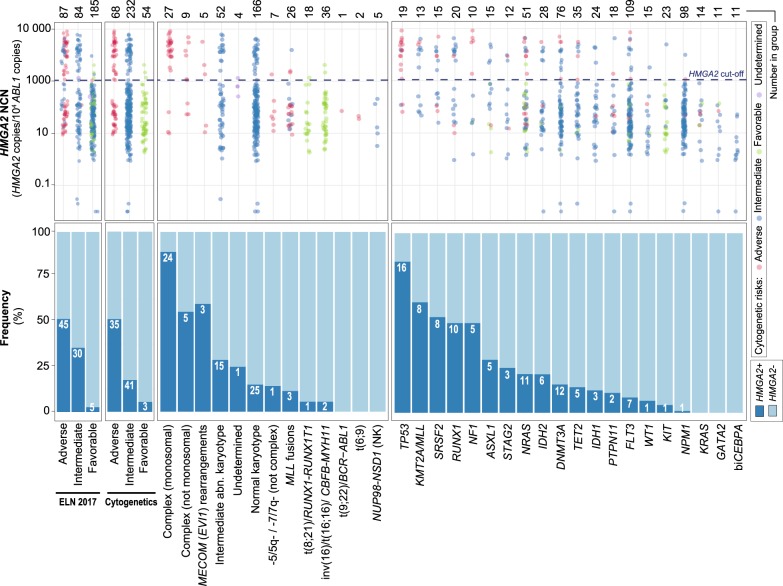
*HMGA2* expression in AML cytogenetic and mutation subgroups. Upper panel. The HMGA2 RT-qPCR test shows a large range of expression values among cytogenetic and mutation subgroups of the training cohort. *HMGA2* expression levels evaluated by this test were normalized on the *ABL1* control gene and expressed as NCN. The dotted line represents the assay cutoff established at 1100 NCN. Bottom panel. Frequency of patients classified in each subgroup according to the *HMGA2* expression profile. Numbers in white represent *HMGA2*+ patients. ELN 2017 and cytogenetic risk subgroups were evaluated in the training cohort (*n* = 358), mutations were obtained by RNA sequencing in 263 samples of the training cohort. Abn., abnormal; bi*CEBPA*, biallelic *CEBPA* mutations; ELN: European LeukemiaNet; *HMGA2*+ , high expression (≥1100 NCN); *HMGA2*−, low expression (<1100 NCN); NCN, Normalized Copy Numbers; NK, normal karyotype

We developed and validated a HMGA2 RT-qPCR test in three independent AML patient cohorts (Leucegene, NCRI AML17 and Australian cohorts) and confirmed the bimodal expression pattern of *HMGA2* (Fig. 1 and Supplementary Figure S5). We observed a high correlation between these results and those found by RNA sequencing or droplet digital PCR as well as a large range of expression values (Supplementary Figure S6; Fig. 2 upper panel). Using ROC curves, the cutoff for the RT-qPCR test was optimized and established at 1100 NCN in the training cohort.^23^ Samples with expression levels ≥ 1100 NCN are hereafter referred to as *H+* and those with expression levels < 1100 NCN as *H−*.

In the training cohort, the HMGA2 test showed high reproducibility, robustness, and specificity (Supplementary Table S7). Inter-laboratory test validation was performed at the King’s College University of London laboratory, using 263 AML samples from patients of the NCRI AML17 trial.

### *HMGA2* expression profile in relation to age and genetics

In the training cohort, 38 of 123 (30.9%) older patients (≥60 years) were *H* *+* compared with 42 of 235 (17.9%) younger patients (<60 years) (*P* = 0.007) (Table 1). *H* + status was more frequent in the adverse cytogenetic risk category: 35 patients of 68 (51.5%) were *H* *+* , and in the 2017 ELN adverse-risk group: 45 patients of 87 (51.7%) were *H* *+* . Similar to RNA-sequencing results, most patients with complex and monosomal karyotype (88.9% *H* *+* , 24 of 27) or with *TP53* mutations (84.2% *H* *+* , 16 of 19) were *H* + (Fig. 2). Among the 232 patients in the intermediate cytogenetic risk category, 41 (17.7%) were positive for *HMGA2*. Of those, only a low proportion were *NPM1* (4.9% vs 66.0% in *H*−; *P* < 0.001) or *FLT3*-ITD mutated (22.0% vs 42.4% in *H*−; *P* = 0.021) (Table 1). In the favorable cytogenetic risk category (t(8;21) and inv(16)), only 3 of 54 patients (5.6%) were *H* + (Table 1; Fig. 2). All biallelic *CEBPA* mutated samples were *H*− (Fig. 2). In the validation cohort, high *HMGA2* expression levels were detected in 72 of 214 (33.6%) intermediate cytogenetic risk patients and 24 of 35 (68.6%) adverse cytogenetic risk patients with a lower frequency in *NPM1* (22.2% vs 58.0% in *H*−; *P* < 0.001) or *FLT3*-ITD (12.1% vs 34.4% in *H−*; *P* < 0.001) mutated samples (Table 1).

### HMGA2 test is powerful to predict clinical outcomes in AML

**Fig. 3:**
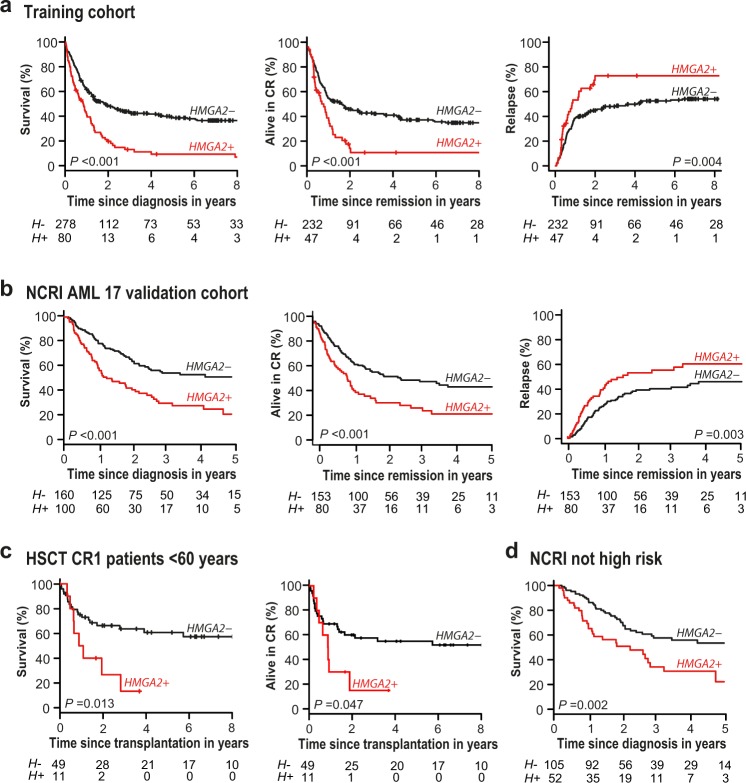
High *HMGA2* expression is associated with poor clinical outcomes in AML. From left to right for **a** the training cohort, **b** NCRI AML17 validation cohort: overall survival (OS), relapse-free survival (RFS), and cumulative incidence of relapse (CIR) curves according to high expression levels of *HMGA2* (*HMGA2* + , *H* *+* ) compared with low expression levels (*HMGA2*−, *H*−). **c** OS and RFS curves for patients ( < 60 years) of the training cohort transplanted in first complete remission (*n* = 60). **d** OS curve for patients of the NCRI AML17 cohort not classified in the high-risk category (*n* = 157). The NCRI high-risk category is defined in the statistical methods section in the Supplementary Information.^30,31^ The *P* values were obtained by the log-rank test for comparison of OS and RFS curves and by Gray’s test for CIR curves

In the training cohort, compared with *H*− patients, *H* + patients had lower CR frequency (58.8% vs 83.4%; *P* < 0.001) (Table 1), worse 3-year OS (13.2% vs 43.5%; *P* < 0.001) and RFS (10.8% vs 44.2%; *P* < 0.001), and a higher 3-year CIR (72.9% vs 48.1%; *P* *=* 0.004) (Fig. 3a; Supplementary Table S10). Univariate analyses showed the strong effect of *H* + as well as age and cytogenetics, for all these clinical outcomes (Supplementary Table S11). Among the 308 patients with ≥3 years of follow-up, only 7 of 72 *H* + patients were still alive at 3 years compared with 87 of 236 *H*− patients (9.7% vs 36.9%; *P* < 0.001) (Supplementary Figure S7).

**Fig. 4:**
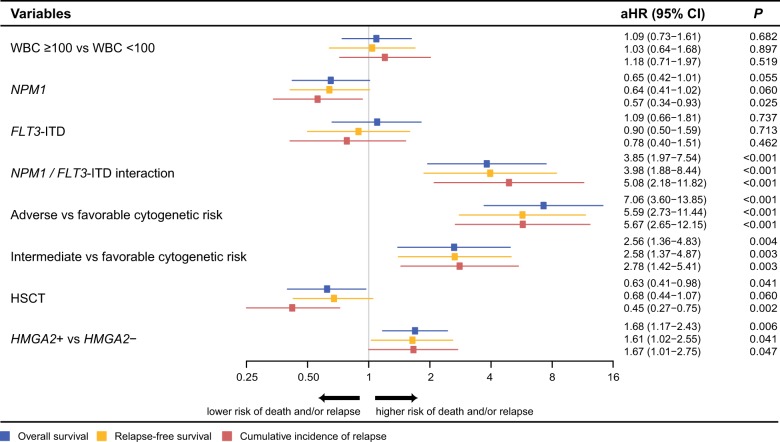
*HMGA2* is an independent prognostic factor of poor outcome in AML. Forest plot for multivariable analyses of overall survival, relapse-free survival and cumulative incidence of relapse in the training cohort. aHR, adjusted hazard ratio; CI, confidence intervals; *HMGA2* *+* , high expression ( ≥ 1100 NCN); *HMGA2-*, low expression (<1100 NCN); HSCT, allogeneic hematopoietic stem cell transplantation; ITD, internal tandem duplication; WBC, white blood cell counts (×10^9^/l). As the non-linear effect of age at diagnosis is represented jointly by the two coefficients (linear and quadratic), the interpretation of each coefficient separately is not appropriate and not shown in the figure

Multivariable analyses, adjusted for age, WBC counts, HSCT as a time-dependent variable (except for CR prediction), cytogenetic risk and *NPM1* and *FLT3*-ITD mutation status, revealed that *H* *+* was independently associated with a significantly higher probability of primary refractory disease (adjusted Odds ratio = 3.08, (95% confidence interval (CI), 1.44–6.59), *P* = 0.004), worse OS (adjusted Hazard ratio = 1.68, (95% CI, 1.17–2.43), *P* = 0.006) and RFS (aHR = 1.61, (95% CI, 1.02–2.55), *P* = 0.041) and a higher CIR (aHR = 1.67, (95% CI, 1.01–2.75), *P* = 0.047) (Table 2; Fig. 4). Importantly, among the 263 sequenced patients of the Leucegene prognostic cohort (Fig. 1), even after having adjusted for the 2017 ELN poor risk mutations (*TP53*, *ASXL1*, and *RUNX1*), *HMGA2* remained a strong predictor for poor response to induction chemotherapy (aOR = 4.03, (95% CI, 1.55–10.4), *P* = 0.004) (Table 2) and worse OS (aHR = 1.73, (95% CI, 1.06–2.84), *P* = 0.030) (Supplementary Table S12). Interestingly, after adjusting for *HMGA2*, *TP53* mutations lost their statistical significance in the OS model (Supplementary Table S12).

**Table 2:** Results of multivariable analysis for complete remission in the training cohort (*n* = 358) and in the sequenced cohort (*n* = 263)

Variables	aOR (95% CI)		*P*
Training cohort	WBC ≥ 100 vs WBC < 100	1.26 (0.58–2.73)	0.559
	*NPM1*	0.51 (0.18–1.46)	0.208
	*FLT3*-ITD	0.54 (0.18–1.66)	0.284
	*NPM1/FLT3*-ITD interaction	8.26 (1.82–37.40)	0.006
	Adverse vs favorable cytogenetic risk	5.48 (1.38–21.80)	0.016
	Intermediate vs favorable cytogenetic risk	2.30 (0.60–8.82)	0.226
	*HMGA2*+ vs *HMGA2*−	3.08 (1.44–6.59)	0.004
Sequenced cohort	WBC ≥ 100 vs WBC < 100	1.41 (0.63–3.2)	0.406
	*NPM1*	0.4 (0.11–1.43)	0.158
	*FLT3*-ITD	0.41 (0.1–1.72)	0.226
	*NPM1/FLT3*-ITD interaction	11.9 (1.86–76.6)	0.009
	Adverse vs favorable cytogenetic risk	6.54 (1.54–27.9)	0.011
	Intermediate vs favorable cytogenetic risk	2.42 (0.58–10.1)	0.228
	*HMGA2*+ vs *HMGA2*-	4.03 (1.55–10.4)	0.004
	*ASXL1*	0.53 (0.11–2.66)	0.442
	*RUNX1*	1.62 (0.47–5.65)	0.446
	*TP53*	0.41 (0.1–1.67)	0.215

*aOR* adjusted odds ratio, *CI* confidence intervals, *HMGA2+* high expression (≥1100 NCN), *HMGA2*− low expression (<1100 NCN), *ITD* internal tandem duplication, *WBC* white blood cells (× 10^9^/l)

As the non-linear effect of age at diagnosis is represented jointly by the two coefficients (linear and quadratic), the interpretation of each coefficient separately is not appropriate. See statistical methods (Supplementary Information) for description of the adjusted effect of age at diagnosis

### HMGA2 test is clinically useful in intermediate genetic risk patients

**Fig. 5:**
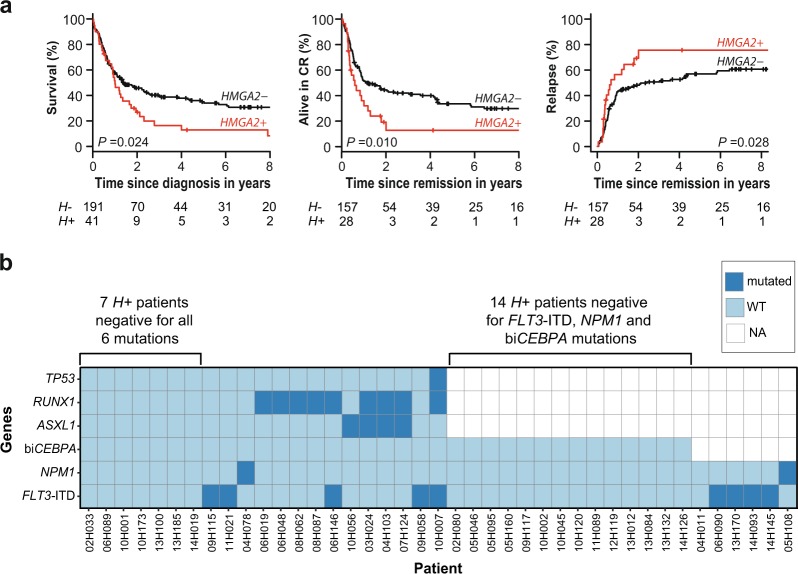
Utility of the HMGA2 test in intermediate cytogenetic risk AML patients. **a** From left to right for patients of the training cohort classified in the intermediate cytogenetic risk category: overall survival (OS), relapse-free survival (RFS) and cumulative incidence of relapse (CIR) curves according to high expression levels of *HMGA2* (*HMGA2* + , *H* *+* ) compared with low expression levels (*HMGA2*−, *H*−). The *P* values were obtained by the log-rank test for comparison of OS and RFS curves and by Gray’s test for CIR curves. **b** Results for the six prognostically informative AML mutations (*FLT3*-ITD, *NPM1*, biallelic *CEBPA*, *ASXL1*, *RUNX1*, and *TP53*) in 41 *H* *+* patients of the training cohort classified in the intermediate cytogenetic risk category are shown. Dark blue squares, presence of mutation; light blue squares, absence of mutation (WT); white squares, sample not tested or not sequenced for this mutation (NA); *H* + , high expression levels of *HMGA2* (≥1100 NCN)

The prognostic value of *HMGA2* expression was also evaluated in the intermediate cytogenetic risk category. In this subgroup, a positive HMGA2 test (41 of 232 patients) also predicted poor clinical outcomes (OS: 17.3% vs 40.2% for *H−* patients, *P* = 0.024; RFS: 12.8% vs 42.0%, *P* = 0.010; CIR: 75.6% vs 50.6%, *P* *=* 0.028) (Fig. 5a and Supplementary Table S10). Importantly, among these *H* + patients, seven were negative for the six prognostically informative AML mutations (*FLT3*-ITD, *NPM1*, biallelic *CEBPA*, *ASXL1*, *RUNX1*, and *TP53*) (Fig. 5b). Moreover, in 14 additional *H* + patients negative for *FLT3*-ITD, *NPM1*, and biallelic *CEBPA* mutations, and for which mutation profiling of *ASXL1*, *RUNX1*, and *TP53* genes was not available in the clinical laboratory, the HMGA2 test could have been useful to identify poor risk patients (Fig. 5b). RNA-sequencing data were available for 165 intermediate cytogenetic risk patients: mutations in *ASXL1* and/or *RUNX1* genes were detected in 22 patients. Only 2 of these 165 patients had *TP53* mutations and were excluded from the multivariable analyses. Even after having adjusted for *FLT3*-ITD, *NPM1*, biallelic *CEBPA*, *ASXL1* and *RUNX1* mutations, the WBC count and HSCT as a time-dependent variable, the significant independent impact of *H* *+* for survival and relapse prediction was further confirmed (OS: aHR = 2.38, (95% CI, 1.26–4.50), *P* = 0.008; RFS: aHR = 2.67, (95% CI, 1.24–5.77), *P* = 0.012; CIR (aHR = 2.61, (95% CI, 1.13–6.05), *P* = 0.025) (Supplementary Table S13).

### HMGA2 test in transplanted patients

Among 60 younger patients who underwent allo-HSCT in first CR, including 42 intermediate-risk patients, *H* + was highly predictive of poor OS (3-year OS: 13.3% vs 63.6%, *P* = 0.013) and RFS (3-year RFS: 15.0% vs 57.6%, *P* = 0.047) (Fig. 3c) and appeared to be associated with a higher CIR (3-year CIR: 65.0% vs 27.7%, *P* = 0.064) (Supplementary Figure S8; Supplementary Table S10). However, the number of transplanted patients was too small for multivariable analysis.

### HMGA2 test also adds prognostic value in the 2017 ELN adverse-risk category

We next studied whether the HMGA2 test could improve prognostic assessment in AML patients classified according to the 2017 ELN genetic risk stratification.^1^ We found that 45 out of 87 ELN adverse-risk patients (51.7%) (Table 1, Supplementary Table S14) were positive for the HMGA2 test and had a significantly worse survival (Supplementary Figure S9, right panel, red curve). In this patient subgroup (ELN adverse-*H* *+* ), representing 12.6% of the entire AML training cohort, no patients were long-term survivors. In contrast, the survival of *H*− patients classified as adverse risk by the ELN risk stratification was similar to that of ELN intermediate-risk patients (Supplementary Figure S9, right panel, yellow and green curves). Importantly, among the 45 *H* *+* patients, eight samples harbored mutations in *RUNX1* and/or *ASXL1* genes (intermediate-risk cytogenetics) and 15 had mutations in *TP53* (Supplementary Table S14). This finding is clinically relevant, especially if screening for these poor risk mutations is not readily available.

### HMGA2 test validation in the NCRI AML17 cohort

To validate the ability of *HMGA2* expression to enhance risk stratification in an independent cohort, the prognostic value of *H* *+* was assessed in the UK NCRI AML17 cohort using the same RT-qPCR assay and cutoff (Table 1). Consistent with our findings, *H* *+* was a strong predictor of a lower frequency of CR (70% vs 85.6%, *P* *=* 0.002, Table 1), poor survival (5-year OS: 21% vs 51%, *P* *<* 0.001) and a higher risk of relapse (5-year RFS: 21% vs 44%, *P* *<* 0.001 and 5-year CIR: 60% vs 46%, *P* = 0.003) in the validation cohort (Table 3, Fig. 3b). Multivariable logistic and Cox regression analyses were used to examine the effect of *HMGA2* expression adjusted for these known prognostic variables: age, log WBC count, secondary disease, WHO/ECOG performance status, presence of adverse cytogenetics, *FLT3*-ITD and *NPM1* mutations. These results confirmed that *H* *+* was significantly and independently associated with lower CR/CRi (CR with incomplete hematologic recovery) frequency (aOR = 3.98, (95% CI, 1.36–11.65), *P* = 0.010), worse OS (aHR = 2.03, (95% CI, 1.36–3.03), *P* < 0.001), and RFS (aHR = 2.06, (95% CI, 1.38–3.08), *P* < 0.001) and a higher CIR (aHR = 2.01 (95% CI, 1.28–3.14), *P* = 0.002) (Table 3). The utility of the HMGA2 test was also evaluated in AML patients classified using a clinical risk score to identify high-risk patients. High-risk disease was defined according to the NCRI multi-parameter risk score, based upon baseline characteristics and response to the first course of induction chemotherapy^30,31^ (detailed in Supplementary Information, Statistical Methods section). Importantly, among the 157 patients not classified in the NCRI high-risk category, 52 (33%) *H* *+* patients had a significantly worse survival than 105 *H*− patients (*P* = 0.002) (Fig. 3d).

**Table 3:** Results of univariate and multivariable analyses for *HMGA2* in the NCRI AML17 validation cohort

Outcome	*HMGA2* *−*	*HMGA2* *+*	Unadjusted OR/HR (95% CI) *P*	Adjusted^a^ OR/HR (95% CI) *P*
	*n* = 160	*n* = 100		
CR and CRi^b^	95.6%	80%	5.05 (2.20–11.6) <0.001	3.98 (1.36–11.65) 0.010
Overall survival	51%^c^	21%^c^	2.33 (1.61–3.36) <0.001	2.03 (1.36–3.03) <0.001
Relapse-free survival	44%^c^	21%^c^	2.13 (1.45–3.13) <0.001	2.06 (1.38–3.08) <0.001
Cumulative incidence of relapse	46%^c^	60%^c^	1.97 (1.28–3.03) 0.002	2.01 (1.28–3.14) 0.002
Cumulative incidence of death	10%^c^	18%^c^	2.87 (1.22–6.75) 0.020	2.29 (0.89–5.87) 0.090
Overall survival censored at transplant	60%^c^	31%^c^	2.70 (1.68–4.34) <0.001	2.00 (1.18–3.39) 0.010

*CI* confidence intervals, *HMGA2*− low expression (<1100 NCN), *HMGA2*+ high expression (≥1100 NCN), *HR* hazard ratio, *OR* odds ratio

^a^Variables included in the multivariable models are: age, log white blood cell count, secondary disease, WHO/ECOG performance status, the presence of adverse cytogenetics, *FLT3*-ITD, and *NPM1* mutations

^b^Complete remission (CR) and complete remission with incomplete hematologic recovery (CRi) excluding induction deaths

^c^Clinical end-points at 5 years

## Discussion

*HMGA2* encodes a member of the HMGA family of proteins implicated in chromatin remodeling and transcription regulation. It is overexpressed in many human solid tumors and its upregulation was thought to be potentially associated with tumor progression and poor prognosis.^32,33^ This study reports the strong negative prognostic impact of *HMGA2* overexpression in AML, thus justifying the development and validation of a rapid, simple and inexpensive RT-qPCR test, also optimized on the droplet digital PCR platform, which can now be implemented in clinical laboratories. Our findings reveal that high *HMGA2* expression confers a significantly higher probability of primary refractory disease after an anthracycline and cytarabine based induction chemotherapy. Interestingly, in the training cohort, the HMGA2 test also reclassified 17.7% of intermediate cytogenetic risk patients into a poor risk group. These results were confirmed in the validation cohort in which 33% of patients not classified in the NCRI high-risk category were *H* *+* and had a significantly worse survival than *H* *−* patients. This new knowledge could guide clinicians to consider offering more intensive or novel consolidation therapies for these patients.

Data presented in this study also highlight the possibility that *HMGA2* expression status may predict outcome following allo-HSCT, although our study does not have the power to fully address this issue.

Importantly, in a subgroup of ELN adverse genetic risk patients, a positive HMGA2 test could also predict resistance to standard treatments including allogeneic stem cell transplantation. However, these results require further validation in other AML cohorts with comprehensive mutation profiling data and classified according to the 2017 ELN genetic risk categories. Future prospective studies will determine if specific therapeutic strategies such as investigational new drugs or novel transplantation methods can improve the clinical outcome of *HMGA2* positive patients.

Although age, mutations, and cytogenetic characteristics affect patient survival in AML, we demonstrate that expression of a single gene, *HMGA2*, is an independent prognostic factor in multivariable analyses in two independent AML cohorts. Moreover, *HMGA2* appears to integrate the negative prognostic value conferred by complex karyotype and several poor risk mutations and could simplify prognostic assessment of positive cases. However, the test did not capture all poor prognosis patient subgroups. For example, *MLL* rearrangements and the poor prognostic *NPM1* + *FLT3*-ITD + *DNMT3A* + subset^3^ (~ 7% and ~ 12.5% in the Leucegene cohort, respectively) were frequently associated with low expression levels of *HMGA2*. Based on these findings, we propose a new algorithm integrating the HMGA2 test in current strategies for AML prognostic assessment (Supplementary Figure S10). Validation of this algorithm in clinical trials is warranted.

In conclusion, this study showed that high *HMGA2* expression adds significant independent prognostic value to known clinical and genetic prognostic factors in AML, and is predictive of poor clinical outcomes with standard AML therapies. The HMGA2 test could complement the current AML tests to improve treatment orientation and be integrated in ongoing and future prospective clinical trials studying innovative therapies to increase survival of *HMGA2* positive AML patients.

## Electronic supplementary material

Supplementary information

Supplementary material: clinical, qPCR, and mutational data.
