# Correction: High expression of HMGA2 independently predicts poor clinical outcomes in acute myeloid leukemia

**DOI:** 10.1038/s41408-019-0190-z

**Published:** 2019-02-28

**Authors:** Miriam Marquis, Cyrielle Beaubois, Vincent-Philippe Lavallée, Michal Abrahamowicz, Coraline Danieli, Sébastien Lemieux, Imran Ahmad, Andrew Wei, Stephen B. Ting, Shaun Fleming, Anthony Schwarer, David Grimwade, William Grey, Robert K. Hills, Paresh Vyas, Nigel Russell, Guy Sauvageau, Josée Hébert

**Affiliations:** 1 The Quebec Leukemia Cell Bank, Research Centre, Maisonneuve-Rosemont Hospital, Montréal, Canada; 2 The Leucegene project at Institute for Research in Immunology and Cancer, Université de Montréal, Montréal, Canada; 3 Division of Hematology-Oncology, Maisonneuve-Rosemont Hospital, Montréal, Canada; 4 Epidemiology and Biostatistics Department, McGill University, Montréal, Canada; 5 Department of Computer Science and Operations Research, Université de Montréal, Montréal, Canada; 6 Department of Medicine, Faculty of Medicine, Université de Montréal, Montréal, Canada; 7 Department of Haematology, Alfred Hospital, Melbourne, Australia; 8 Australian Centre for Blood Diseases, Monash University, Melbourne, Australia; 9 Faculty of Medicine, Nursing and Health Sciences, Monash University, Melbourne, Australia; 10 Department of Haematology, Eastern Health, Box Hill Hospital, Melbourne, Australia; 11 Cancer Genetics Laboratory, Department of Medical and Molecular Genetics, King’s College London, London, UK; 12 UK National Cancer Research Institute (NCRI) Haematological Oncology Clinical Studies Group, Cardiff, UK; 13 Centre for Trials Research, Cardiff University School of Medicine, Cardiff, UK; 14 MRC Molecular Haematology Unit, Weatherall Institute of Molecular Medicine and Department of Haematology, University of Oxford and Oxford University Hospitals NHS Trust, Oxford, UK; 15 NIHR Oxford Biomedical Research Centre, Oxford, UK; 16 Centre for Clinical Haematology, Nottingham University Hospital (City Hospital Campus), Nottingham, UK

**Correction to: Blood Cancer Journal** (2018) **8:** 68

10.1038/s41408-018-0103-6;Published online 19 July 2018

Since the publication of the original article the authors noticed the the affiliation details for Paresh Vyas are incorrect. The correct affiliation details for this author are given below:

- MRC Molecular Haematology Unit, Weatherall Institute of Molecular Medicine and Department of Haematology, University of Oxford and Oxford University Hospitals NHS Trust, Oxford, UK
- NIHR Oxford Biomedical Research Centre, Oxford, UK

