# Supplementary material for: High expression of HMGA2 independently predicts poor clinical outcomes in acute myeloid leukemia
Source: Blood Cancer J. 2018 Jul 19;8(8):68. doi: 10.1038/s41408-018-0103-6 (PMC6066481; doi:10.1038/s41408-018-0103-6)
Supplement: Supplementary file 1 — Supplementary information [file 41408_2018_103_MOESM1_ESM.pdf]

SUPPLEMENTARY INFORMATION

High expression of *HMGA2* independently predicts poor clinical outcomes in acute myeloid leukemia

Marquis M., *et al.*

TABLE OF CONTENTS

METHODS **4**

Methods for cryopreservation and storage of leukemia cells 4

Methods for *NPM1*, *FLT3*-ITD and biallelic *CEBPA* mutation testing 4

Amplification and analysis of *HMGA2* transcripts 4

Plasmid standard curves 5

Analytical validation of the HMGA2 RT-qPCR test 6

Droplet digital PCR experiments 6

Evaluation of the HMGA2 RT-qPCR test in other cohorts 7

STATISTICAL METHODS **8**

Rationale for sample size for the training cohort 8

Missing data handling 9

Proportional hazards (PH) assumption 9

Age: linear and quadratic effects in multivariable models 9

FIGURES **12**

Figure S1. Overall survival curves in the training cohort and according to age 12

Figure S2. Overall survival according to cytogenetic risk categories in the training cohort 13

Figure S3. Impact of *PAWR* expression levels on overall survival and relapse-free survival in the NCRI AML17 cohort 14

Figure S4. Enrichment in mutations and cytogenetic groups in *HMGA2*+ and *HMGA2*- AML 15

Figure S5. *HMGA2* expression levels evaluated by RT-qPCR in the three tested AML sample cohorts 16

Figure S6. High correlation between *HMGA2* expression values obtained through 3 technologies 17

Figure S7. Flow diagram and status of patients evaluated at 3-year follow-up in the training cohort according to *HMGA2* expression levels 18

Figure S8. Impact of *HMGA2* expression levels on cumulative incidence of relapse curve in *de novo* AML patients aged less than 60 years old and transplanted in first complete remission (n = 60). 19

Figure S9. The 2017 ELN genetic risk stratification and the HMGA2 test. 20

Figure S10. Proposed algorithm for laboratory testing in AML prognostic assessment. 21

TABLES **22**

Table S1. Clinical characteristics of *de novo* AML patients in the training cohort (n = 358) 22

Table S2. Treatment regimens in the training cohort (n = 358) 23

Table S3. Frequencies according to cytogenetic subgroups in the training cohort (n = 358) 24

Table S4. Frequencies according to the WHO 2008 categories in the training cohort (n = 358) 25

Table S5. Clinical characteristics of the Australian cohort (n = 70) 26

Table S6. Primers and probe used for the HMGA2 RT-qPCR test 27

Table S7. Analytical validation of the HMGA2 RT-qPCR test 28

Table S8. Compliance with REMARK guidelines 29
Table S9. Results of multivariable analyses for *PAWR* in the NCRI AML17 validation cohort 31
Table S10. Overall survival, relapse-free survival and cumulative incidence of relapse frequencies at 3 years in the training cohort, the intermediate cytogenetic risk patients and in transplanted patients under 60 years old 32
Table S11. Univariate analysis of complete remission, relapse-free survival, overall survival, and cumulative incidence of relapse in the training cohort 33
Table S12. Results of multivariable analysis for overall survival adjusted for the ELN poor risk mutations *TP53, ASXL1* and *RUNX1* in the sequenced cohort (n = 263) 34
Table S13. Multivariable analysis of complete remission, relapse-free survival, overall survival, and cumulative incidence of relapse in sequenced patients of the training cohort classified in the intermediate cytogenetic risk category (n = 163) 35
Table S14. 2017 ELN genetic risk stratification and *HMGA2* expression levels in the training cohort (n = 358) 36

REFERENCES 37

METHODS

Methods for cryopreservation and storage of leukemia cells

Mononuclear cells were purified from bone marrow, peripheral blood or leukapheresis samples by Ficoll density centrifugation and cryopreserved in liquid nitrogen (DMSO 10%) and in TRIzol reagent (Invitrogen). All samples of the training cohort were collected, prepared and cryopreserved by the Banque de cellules leucémiques du Québec (BCLQ, http://bclq.org/) which is certified by the Canadian Tissue Repository Network (CTRNet). Standard Operating Procedures of the BCLQ are in accordance with the CTRNet guidelines (http://www.ctrnet.ca/resources/operating-procedures).

Methods for *NPM1*, *FLT3*-ITD and biallelic *CEBPA* mutation testing

Before May 2013, *NPM1* mutations were identified as reported by Falini and al[1]. with a modification of the reverse primer for the sequencing reaction (5'-TTTCCATGTCTGACCACCGCTACT-3'). After May 2013, sequencing was replaced by capillary electrophoresis. Genomic DNA was amplified with the primers NPM1-For2-FRG: 5'-TTTTTTTCCAGGCTATTCAAGATC-3', and NPM1-Rev-ADN-HEX: 5'-HEX-TTTCCATGTCTGACCACCGCTA-3'. Reactions of 20 µl contained 100 ng of genomic DNA, 10 ρmol of primers, deoxynucleoside-5'-triphosphates (0.2 mM), 1X PCR buffer with $MgCl_2$ 10x concentrated (Roche), 1.0 units of Taq DNA polymerase 5 U/µl (Roche). After an initial denaturation step at 95°C for 5 minutes, DNA was amplified for 35 cycles at 95°C for 30 seconds, 56°C for 45 seconds, 72°C for 30 seconds, followed by an elongation step at 72°C for 10 minutes. One µl of PCR products was added to 10 µl of a mix containing formamide and GeneScan ROX 400HD internal size standards (ratio of 1:50, Applied Biosystems) and heated at 95°C for 3 minutes followed by 2 minutes at 4°C. Samples were tested on a Thermofisher 3730XL DNA Analyzer.

Biallelic *CEBPA*[2] and *FLT3*-ITD[3] mutations were assessed using previously described methods. For *FLT3*-ITD, allelic ratios ≥ 5% were considered as positive.[4]

Amplification and analysis of *HMGA2* transcripts

Total RNA was extracted from mononuclear cells with TRIzol reagent according to the manufacturer's protocol (Life Technologies). RNA samples were routinely evaluated for integrity using a Bioanalyzer 2100 (Agilent Technologies) and for quantification using a NanoDrop 2000

(Thermo Scientific). Only RNA samples with RNA Integrity Number (RIN) ≥8 were included in this study. One µg of RNA was reverse transcribed into complementary DNA (cDNA) with the QuantiTect Reverse Transcription Kit (Qiagen GmbH, Hilden) in a 20 µl reaction mix according to the manufacturer's protocol. At the end of the reverse transcriptase (RT) reaction, 30 µl of RNase-Free water was added for a total volume of 50 µl. For all tested samples, another 1 µg of RNA was used to prepare a no RT control in order to assess genomic DNA contamination (gDNA negative control). Primers and probe for *HMGA2* (NM_003483.4, NM_003484.1, NM_001300919.1, NM_001300918.1) (Supplementary Table S6) were designed using Primer3 software.[5] The *ABL1* specific primers and probe were reported previously.[6] The qPCR reaction mixture contained 12.5 µl of TaqMan Gene Expression Master Mix (Applied Biosystems), 500 nM of each primer, 200 nM of FAM/ZEN/3' IBFQ probe (IDT), and 5 µl of cDNA (or plasmid dilutions) in a total volume of 25 µl. The PCR conditions were 2 min at 50°C, 10 min polymerase activation at 95°C and 50 cycles of denaturation at 95°C for 15 sec and annealing at 64°C for 35 sec. All samples were run blindly in duplicates on an ABI 7500 instrument (Applied Biosystems, ThermoFisher Scientific). In addition to the tested samples, each analytical run contained the target and control gene plasmid standard curves in duplicates, two positive controls of known concentration and the following negative controls: a reverse transcriptase (RT) negative control (RT-, no RNA at the RT step) and a no template control (no cDNA).[7] The HMGA2 test was also validated on the QuantStudio™ 7 Flex System using the TaqMan Fast Advance Master Mix (Applied Biosystems). The 7500 Software v2.0.5 and the QuantStudio Real-Time PCR Software (Applied Biosystems) were used to visualize amplification curves and calculate copy number values at a threshold of 0.1 and the baseline was set at 3-15 for *HMGA2* and for *ABL1*, used as the control gene. To avoid misleading interpretation of negative samples, a value of 0.01 copy number was added for all individual samples. According to the Europe Against Cancer (EAC) program,[6] samples with *ABL1* cycle-threshold (Ct) values more than 29 were excluded from the study.

Plasmid standard curves

Plasmid standard curves for *HMGA2* and *ABL1* were developed following guidelines of the EAC program[6]. Plasmids containing sequences of *HMGA2* or *ABL1* were obtained through GeneArt™ Gene Synthesis service (Life Technologies). According to the molecular weight of each plasmid (vector backbone pMA-T), 20 µg of the corresponding plasmid was linearized with the ScaI restriction enzyme for 1 h at 37°C. The digested plasmid was serially diluted in a Tris-EDTA solution (pH 8.0) containing 100 ng/µl of yeast tRNA (Sigma-Aldrich). Six successive

dilutions (1000000, 100000, 10000, 1000, 100 and 10 copies/5 µl) were prepared and stored in 100 µl aliquots at -20°C. To generate normalized copy numbers (NCN), *HMGA2* copy numbers were calculated using the plasmid standard curve and then normalized to *ABL1* copy numbers following the EAC program recommendations.[6]

Analytical validation of the HMGA2 RT-qPCR test

An analytical validation adapted from recommendations of the Clinical and Laboratory Standards Institute (CLSI documents EP05-A3, EP09-A3, EP06-A, EP17-A2, EP28-A3)[8-12] was performed to assess the quantitative performance of the HMGA2 RT-qPCR test (Supplementary Table S7). Analytical specificity was evaluated according to the MIQE guidelines.[13] No amplification was detected in any negative control (gDNA, no template control (NTC), RT-). PCR products were analysed using standard agarose gel migration procedure, confirming the correct amplicon size of 91 bp for *HMGA2*. PCR products were also sequenced, confirming amplification of target sequences. The HMGA2 RT-qPCR test efficiency and linearity (n = 41 independent experiments) were established using 10-fold serial dilutions (10^0 to 10^6 copies) of standard plasmid. The acceptable performance criteria for the test efficiency was between 90 and 110% and for the test linearity, $R^2 \geq 0.98$.[13] Analytical sensitivity (Limit of quantification, LoQ) was established by testing the lowest dilutions of the plasmid standard curves (from 10^3 to 10^0) according to the MIQE guidelines.[13] The LoQ is defined as the lowest dilution at which all replicates are amplified (n = 3/3). The variability, expressed as a percentage of the coefficient of variation (% CV) between replicates, must be equal or lower than 2% (% CV ≤2%). Precision was evaluated for repeatability, intermediate precision and robustness with a 10x2x2 protocol (10 days, 2 runs per day, and 2 replicates per run). Robustness was evaluated for two ABI 7500 systems and 2 MasterMix reagent lots. Acceptable performance criteria for the precision (repeatability, intermediate precision and robustness) should not exceed 15% of the CV.[13, 14] The reportable range (n = 30 independent experiments) of the HMGA2 test was established with 10-fold serial dilutions of standard plasmid.

Droplet digital PCR experiments

Primers and probes used for droplet digital PCR (ddPCR) experiments were the same as for the qPCR assays with the following modification: the *ABL1* probe was a HEX/ZEN/3' IBFQ (IDT) and consequently, assays were run in duplex. The ddPCR reaction mixture contained 10 µl of master mix (Supermix for probes (no dUTP), Bio-Rad), 450 nM of each primer, 250 nM of each probe, and 5 µl of cDNA in a total volume of 20 µl. Each reaction mix was converted to droplets

with the QX200 droplet generator (Bio-Rad). Droplet-partitioned samples were then transferred to a 96-well plate, sealed and cycled in a C1000 deep well Thermocycler (Bio-Rad) using the following cycling protocol: 50 cycles at 95ºC for 30 s (denaturation), 55ºC for 1 min (annealing) and an elongation step of 30 s at 72ºC followed by post-cycling steps of 98ºC for 10 min (enzyme inactivation) and an infinite 8ºC hold. The cycled plate was transferred and read using the QX200 reader (Bio-Rad). The same positive and negative controls as for the qPCR experiments were included in each plate. The QuantaSoft Software v1.7.4 (Bio-Rad) was used to visualize *HMGA2* and *ABL1* copy numbers. To avoid misleading interpretation of negative samples, a value of 0.01 copy number was added for all individual samples.

Evaluation of the HMGA2 RT-qPCR test in other cohorts

The AML samples from Australia (n = 70) were used to confirm the distribution of RT-qPCR values. The experiments were performed at the BCLQ laboratory.

For the UK NCRI AML17 validation cohort, the HMGA2 RT-qPCR tests were performed at Professor David Grimwade's laboratory (Cancer Genetics Laboratory, Department of Medical and Molecular Genetics, King's College London, London, UK) using the same protocol and reagents. Three samples out of 263 had no *HMGA2* expression value and were excluded from analyses.

STATISTICAL METHODS

Rationale for sample size for the training cohort

Sample size was *a priori* determined by the total number of AML patients in the BCLQ project who were diagnosed from 2002 to 2014 and met the study inclusion/exclusion criteria. Therefore, we calculated the statistical power to detect significant ($P < 0.05$) associations between high expression levels of *HMGA2* and each clinical outcome. In the power calculations, we used the actual sample size of the training cohort, the actual data on the observed distribution of the proposed marker and the frequency of the clinical outcomes. We first estimated the power to detect hazard ratio (HR) of HR = 2 for overall survival (OS), relapse-free survival (RFS) and cumulative incidence of relapse (CIR); and OR = 2 for complete remission (CR), where high expression levels of *HMGA2* were expected to be associated with lower probability of CR. Then, when the power was lower than 80% for HR = 2 for OS, RFS and CIR, or OR = 2 for CR, we calculated the minimum detectable HR with an adequate power of 80%. In general, our calculations showed that we have excellent power to detect a moderate risk increase for the 4 primary outcomes: CR (Table 1), RFS (Table 2), OS (Table 3) and CIR (Table 4).

Table 1. First complete remission

Group	No Remission No.	Remission No.	Total	Power OR = 2	* Min detectable OR with 80% Power
HMGA2-	46	232	278		
HMGA2+	33	47	80	0.7685	2.05
Total	79	279	358		

* Minimum detectable OR for a power of 0.8 done only if power related with OR = 2 was below 0.80.

Table 2. Relapse-free survival

Group	Alive with no relapse No.	Relapse or Death No.	Total	Power HR = 2	* Min detectable HR with 80% Power
HMGA2-	97	135	232		
HMGA2+	9	38	47	0.9356	-
Total	106	173	279		

* Minimum detectable HR for a power of 0.8 done only if the power related with HR = 2 was below 0.80.

Table 3. Overall survival

Group	Alive No.	Dead No.	Total	Power HR = 2	* Min detectable HR with 80% Power
HMGA2-	117	161	278		

HMGA2+	12	68	80	0.9909	-
Total	129	229	358		

* Minimum detectable HR for a power of 0.8 done only if the power related with HR = 2 was below 0.80.

Table 4. Cumulative incidence of relapse

Group	Alive No.	Death No.	Relapse No.	Total	Power HR = 2	* Min detectable HR with 80% Power
HMGA2-	97	19	116	232		
HMGA2+	9	7	31	47	0.9356	-
Total	106	27	146	279		

* Minimum detectable HR for a power of 0.8 done only if the power related with HR = 2 was below 0.80.

Missing data handling

In the training cohort, missing data were noted for the following variables: *FLT3*-ITD (n = 25, 6.9%), *NPM1* (n = 25, 6.9%), cytogenetic risk group (n = 4, 1.2%) and WBC counts (n = 4, 1.2%). Due to a technical reason implying data missing at random, those patients were excluded from the multivariable analyses.

In the intermediate cytogenetic risk patient subgroup (n=232), missing data were noted for the following mutations: *ASXL1* (n=67, 28.8%), *RUNX1* (n=67, 28.8%), *TP53* (n=67, 28.8%) and bi*CEBPA* (n=43, 18.5%). Accordingly, multivariable analyses were performed only in the subgroup of sequenced patients (n=163) who had results for the 6 clinically relevant mutations.

Proportional hazards (PH) assumption

Tests based on the flexible extensions of the Cox or Lunn-McNeil models confirmed the validity of the proportional hazards (PH) assumption: for the *HMGA2* marker used in the main analyses, the null PH hypothesis was never rejected ($P > 0.10$ in all analyses, data not shown), indicating that predictive ability of *HMGA2* remained approximately constant over the median follow-up of 6 years.

Age: linear and quadratic effects in multivariable models

To test and - if required - account for possibly non-linear associations between age at diagnosis and the logarithm of either Odds (for logistic regression analyses of CR) or Hazard (for all time-to-event analyses of the other outcomes), we have modeled the adjusted effect of the continuous variable representing age at diagnosis with two terms: linear and quadratic (age-squared). In preliminary analyses, for almost all outcomes and vast majority of multivariable models, the quadratic effect of age improved the outcome prediction – over a simpler model limited to a linear effect of age – in a statistically very significant ($P < 0.01$) and clinically

meaningful way. Therefore, to avoid residual confounding[15] of the adjusted associations with the genetic marker of primary interest, in all final multivariable models, we included the squared term for age at diagnosis (in years), in addition to the linear term.

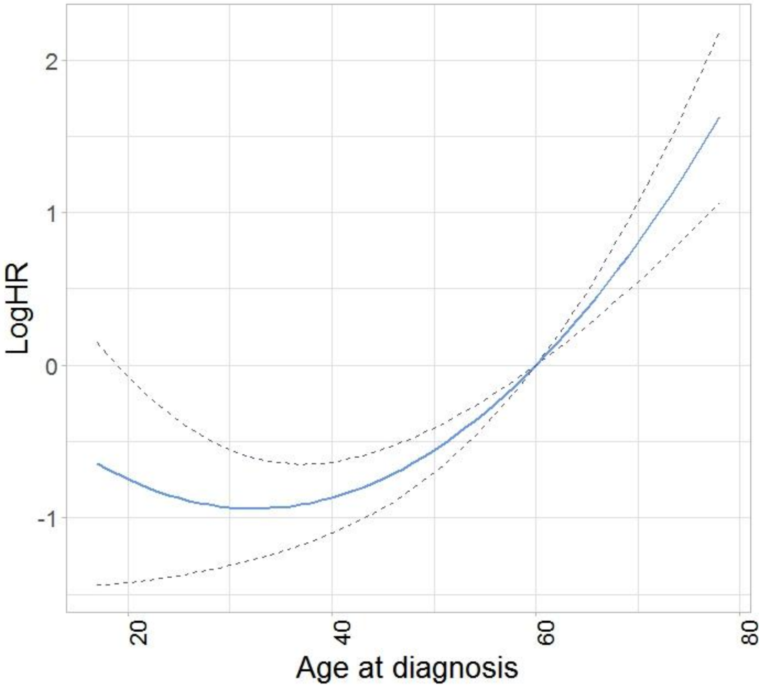

Figure 1. Example of the non-linear effect of age at diagnosis

Results of Cox analysis for overall survival: logarithm of the adjusted Hazard Ratio, for different ages at diagnosis (horizontal axis), relative to a 60 years old subject, with pointwise 95% confidence intervals (dotted curves). This figure shows how the risk of death changes with increasing age at diagnosis. Younger subjects, diagnosed from 20 to 50 years old have the lowest risk, and in this age range, age is not associated with an increased risk of death. In contrast, death risk increases steeply when age at diagnosis increases from 50 to 80 years old.

Statistical methods for the NCRI AML17 validation cohort

Normalised *HMGA2* and *PAWR* values were dichotomised at 1100 and 950 respectively, based on the cut-offs derived from the original analyses of the training cohort. Endpoints were as defined for the training cohort. Univariate analyses were performed using Mantel-Haenszel, and log rank tests. Multivariable logistic and Cox regression analyses were used to examine the effect of the 2 genes investigated adjusted for the known prognostic variables of age, log white blood cell count, secondary disease, WHO/ECOG performance status, the presence of adverse cytogenetics, *FLT3*-ITD and *NPM1* mutations. All adjusted Odds and Hazard Ratios are

reported with 95% confidence intervals; survival estimates are at 5 years. High risk disease was defined according to the NCRI multi-parameter risk score, based upon baseline characteristics and response to course 1, and defined as follows:

0.01325*age (in years) + 0.16994*sex (1 = male, 0 = female) + 0.22131*diagnosis (1 = *de novo*, 2 = secondary) + 0.65082*cytogenetics (1 = favourable, 2 = intermediate, 3 = adverse) + 0.19529*status post C1 (1 = CR, 2 = PR, 3 = NR) + 0.00169* WBC (x 10^9/l).

High risk is defined as a risk score over 2.6667 or a *FLT3*-ITD mutation without an accompanying *NPM1* mutation.[16, 17]

FIGURES

Figure S1. Overall survival curves in the training cohort and according to age

Kaplan-Meier curves for overall survival (OS) (**a**) in the training cohort (n = 358) and (**b**) in the training cohort according to age: 17 to 59 years old (n = 235) vs ≥ 60 years patients (n = 123). The *P* value for comparison of overall survival curves was obtained using the log-rank test.

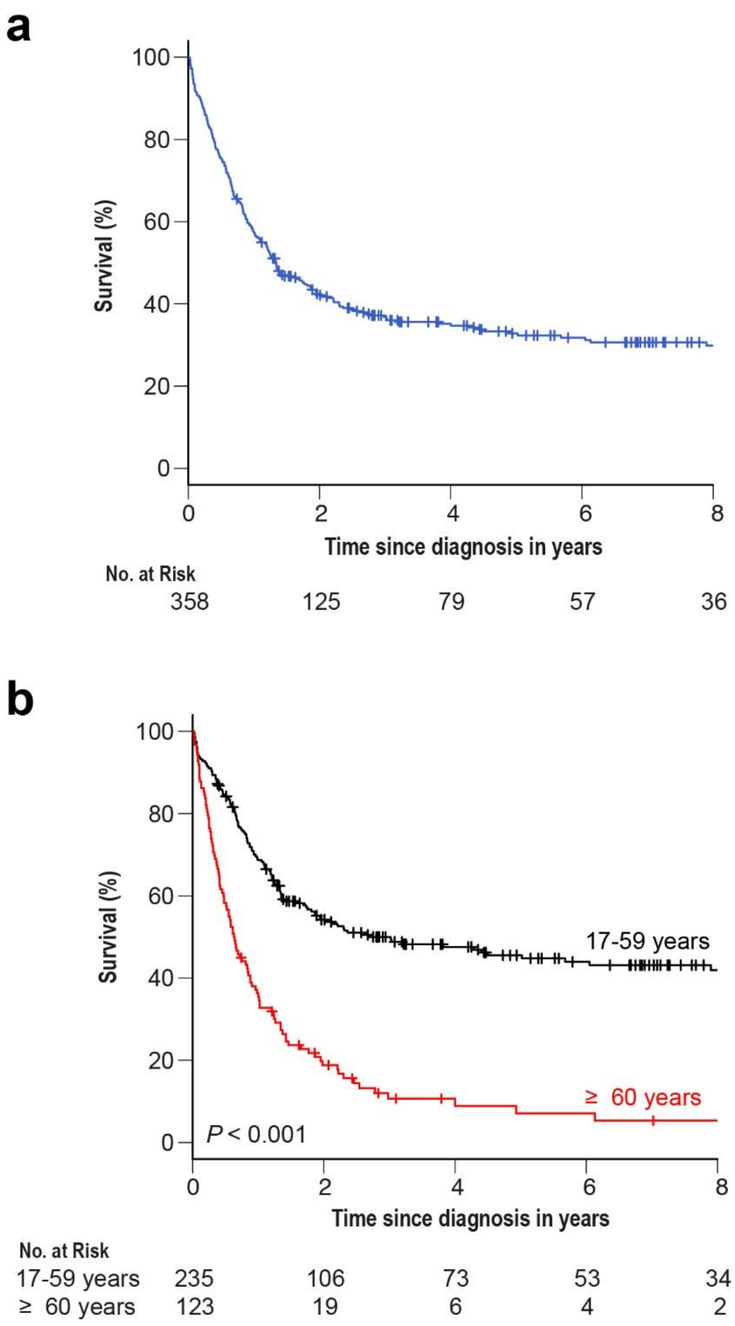

Figure S2. Overall survival according to cytogenetic risk categories in the training cohort

Kaplan-Meier curves for overall survival (OS) of patients classified into the favorable, intermediate and adverse cytogenetic risk groups (**a**) OS curves for all patients in the training cohort, except 4 with an undetermined karyotype (n = 354). (**b**) OS curves for patients under 60 years old in the training cohort, except 3 with an undetermined karyotype (n = 232). The *P* values for comparison of OS curves were obtained using the log-rank test.

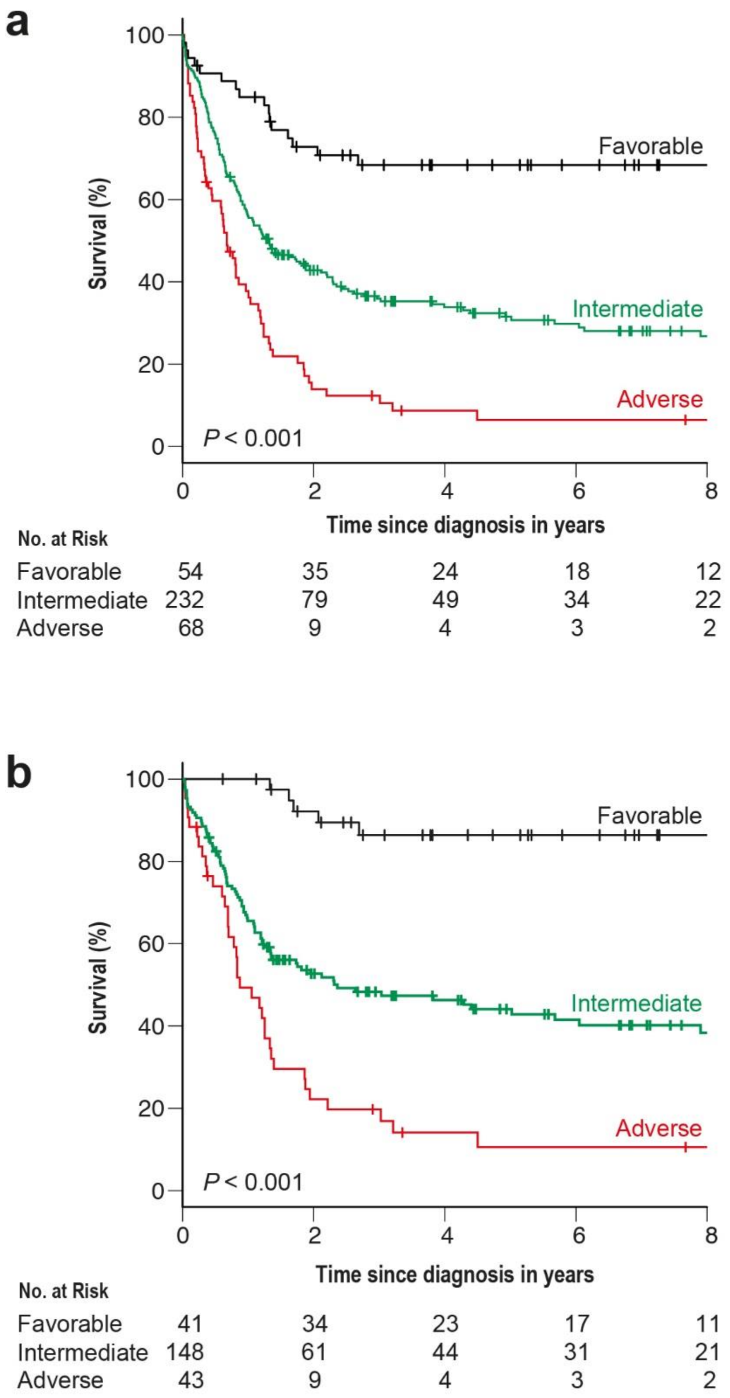

Figure S3. Impact of *PAWR* expression levels on overall survival and relapse-free survival in the NCRI AML17 cohort

Kaplan-Meier curves for (**a**) overall survival and (**b**) relapse-free survival in the NCRI AML17 cohort

a

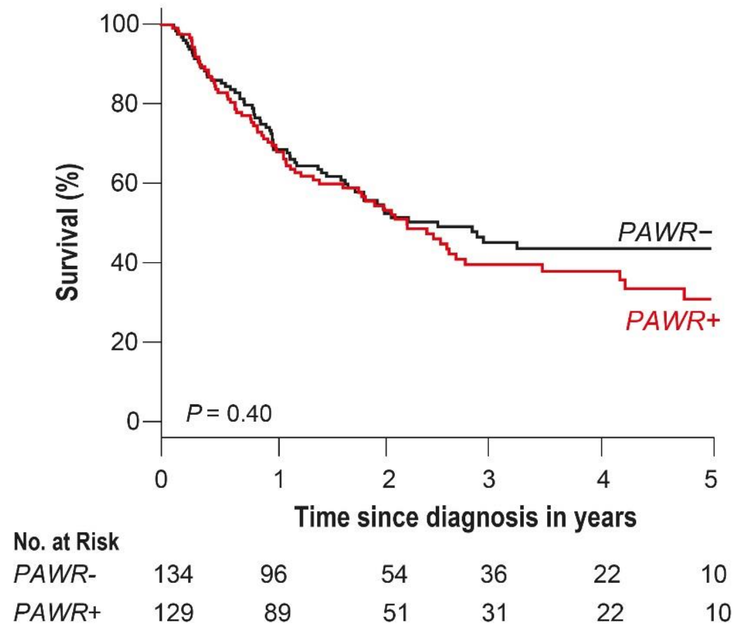

b

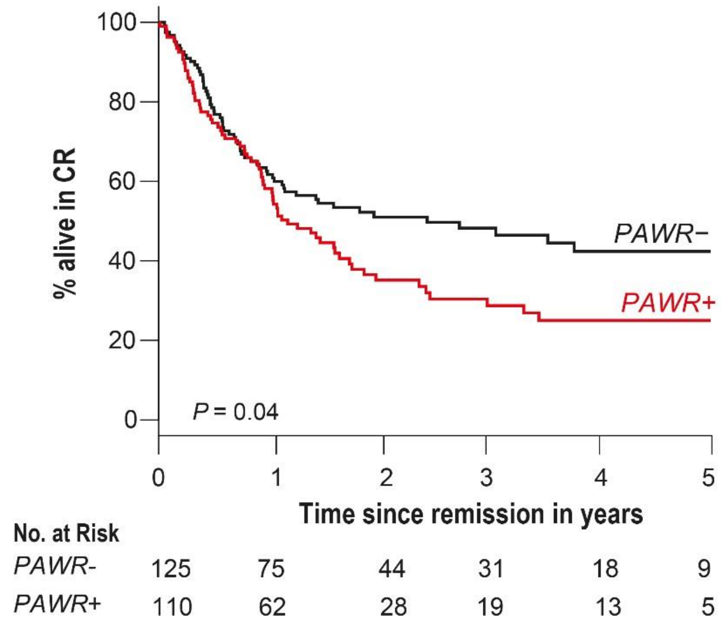

Figure S4. Enrichment in mutations and cytogenetic groups in *HMGA2+* and *HMGA2-* AML

Enrichment in mutations and cytogenetic groups in AML with low (black dots, *HMGA2-*) and high (blue dots, *HMGA2+*) *HMGA2* expression levels, defined by expression levels below or above the 75th percentile in the Leucegene full cohort (430 RNA-sequenced AML samples, detailed in Figure 1). Top enriched genes are labelled. NK, normal karyotype.

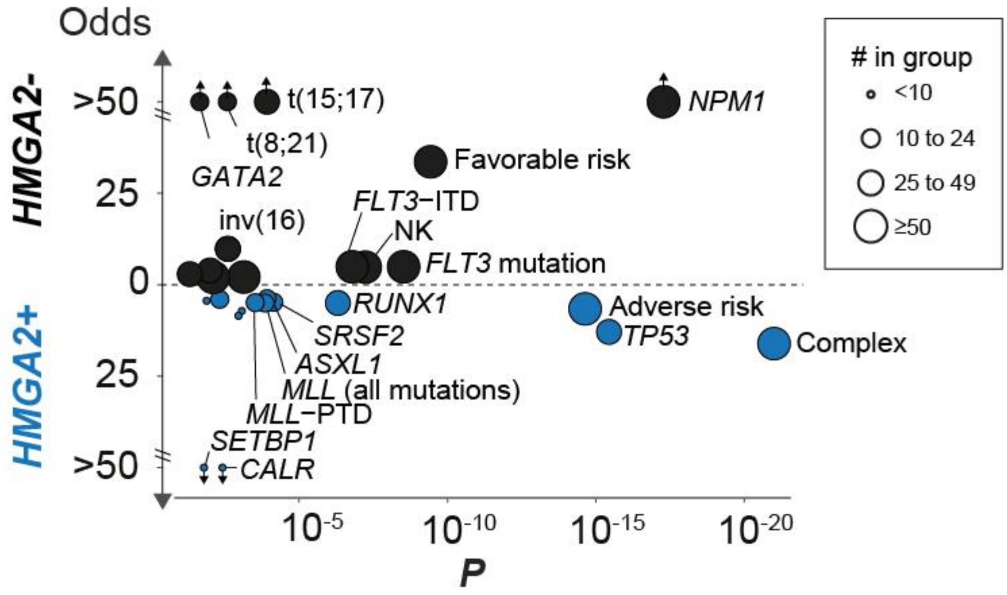

Figure S5. *HMGA2* expression levels evaluated by RT-qPCR in the three tested AML sample cohorts

(**a**) Comparison of density curves in log10 (NCN, Normalized Copy Numbers) between the training cohort, the NCRI AML17 validation cohort and the Australian cohort. The test cut-off (1100 NCN) is represented by a dotted line. (**b**) Distribution of NCN values for *HMGA2* in each cohort.

a

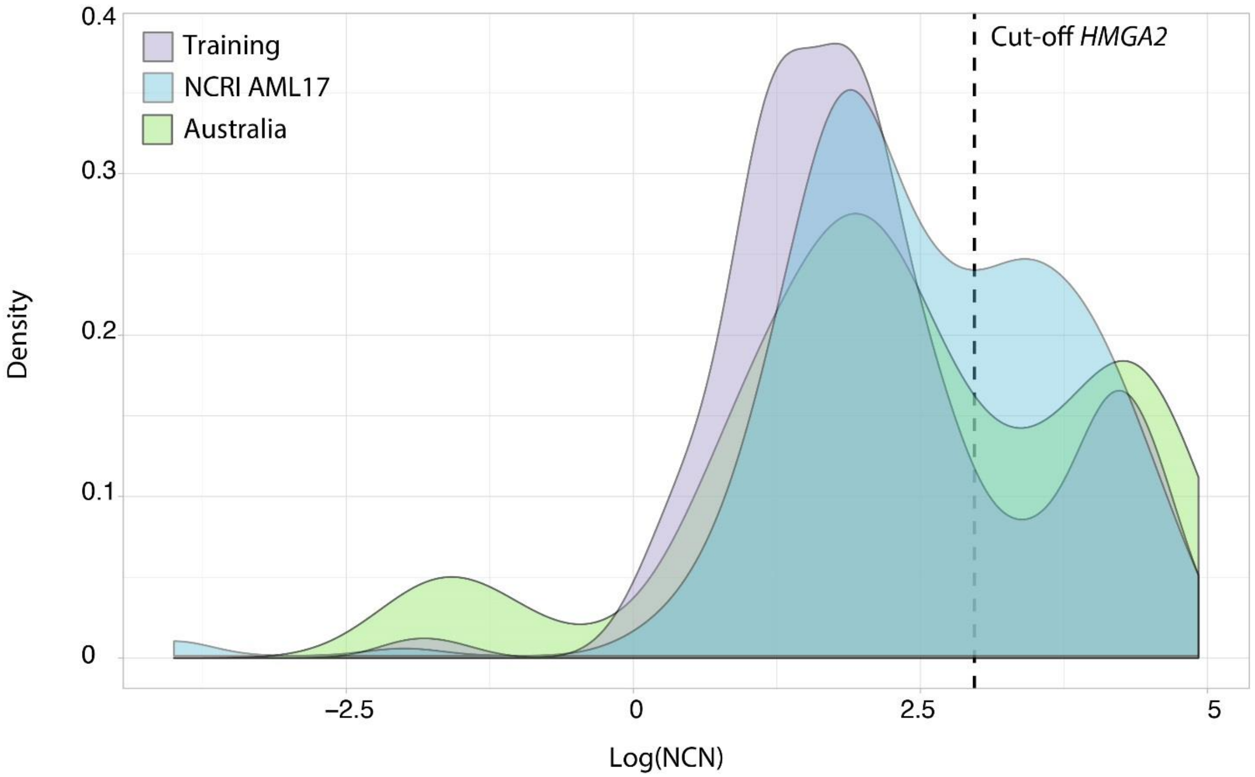

b

Cohort	Mean	SD	0%	25%	50%	75%	100%	n
**Training**	4287.95	11307.74	0.01	17.27	78.84	630.14	83594.89	358
**NCRI AML17**	4478.65	10559.22	0.00	55.51	303.89	3288.23	67689.74	260
**Australia**	7691.15	14462.45	0.01	23.30	153.52	5696.10	58772.21	70

Figure S6. High correlation between *HMGA2* expression values obtained through 3 technologies

(**a**) RNA-Seq (RPKM) *vs* RT-qPCR (NCN) in the 263 sequenced AML samples of the training cohort. (**b**) Droplet digital PCR (NCN) *vs* RT-qPCR (NCN) in 340 AML samples of the training cohort. NCN, Normalized Copy Numbers.

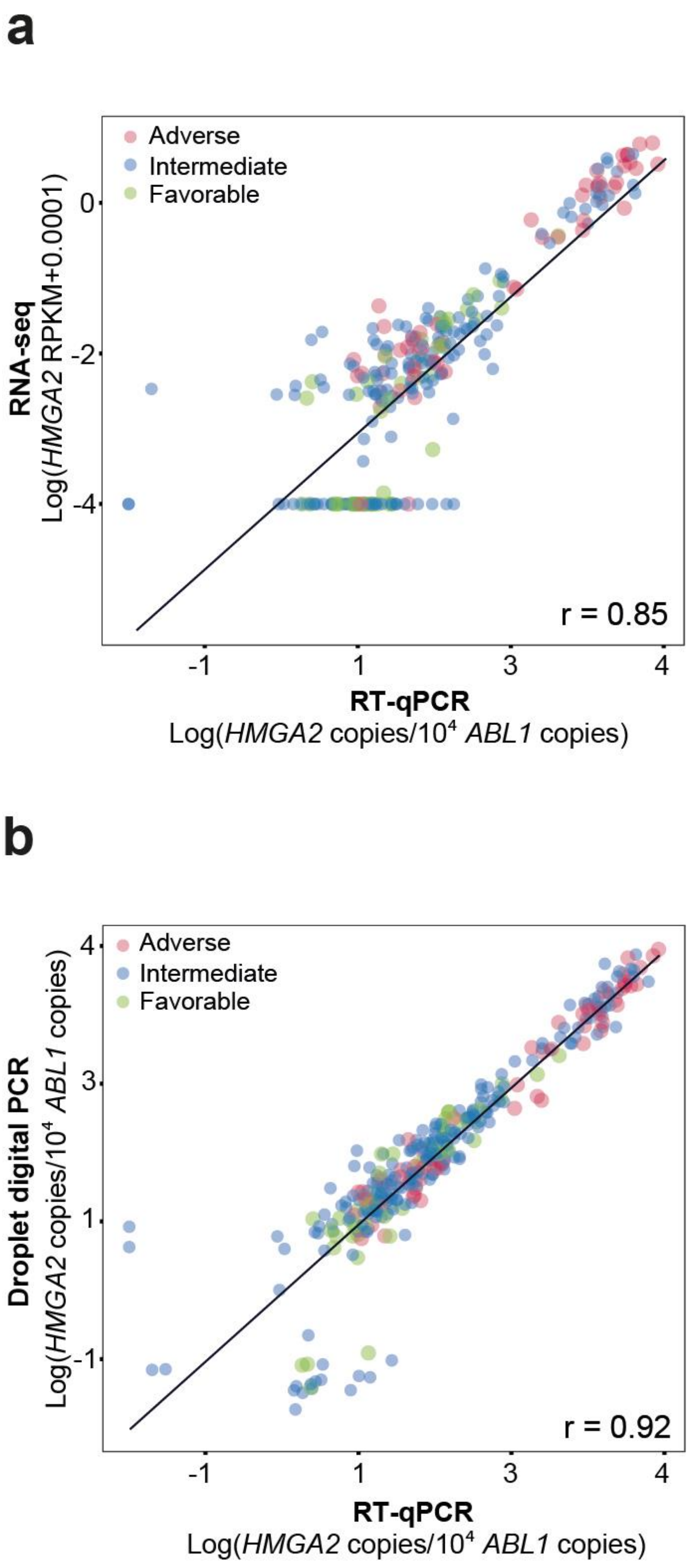

Figure S7. Flow diagram and status of patients evaluated at 3-year follow-up in the training cohort according to *HMGA2* expression levels

(**a**) Flow diagram of the 308 patients having a follow-up of at least 3 years. (**b**) Status at 3 years. Patients who were alive at their last follow-up but who did not complete at least 3 years of follow-up were excluded from these analyses. *HMGA2+*, high expression (≥1100 NCN); *HMGA2-*, low expression (<1100 NCN).

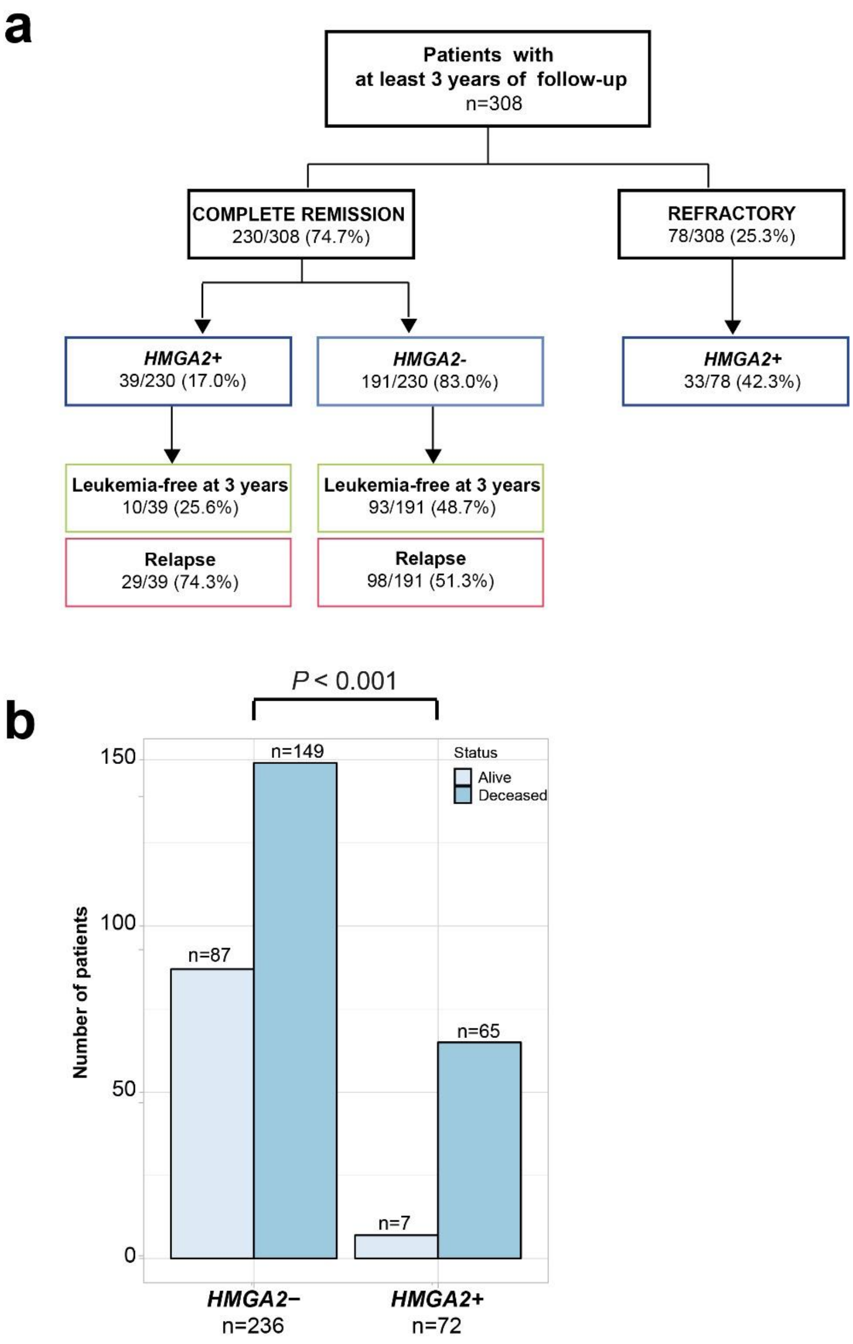

Figure S8. Impact of *HMGA2* expression levels on cumulative incidence of relapse curve in *de novo* AML patients aged less than 60 years old and transplanted in first complete remission (n = 60).

HMGA2+, high expression (≥1100 NCN); *HMGA2-*, low expression (<1100 NCN). The *P* value was obtained using Gray's test.

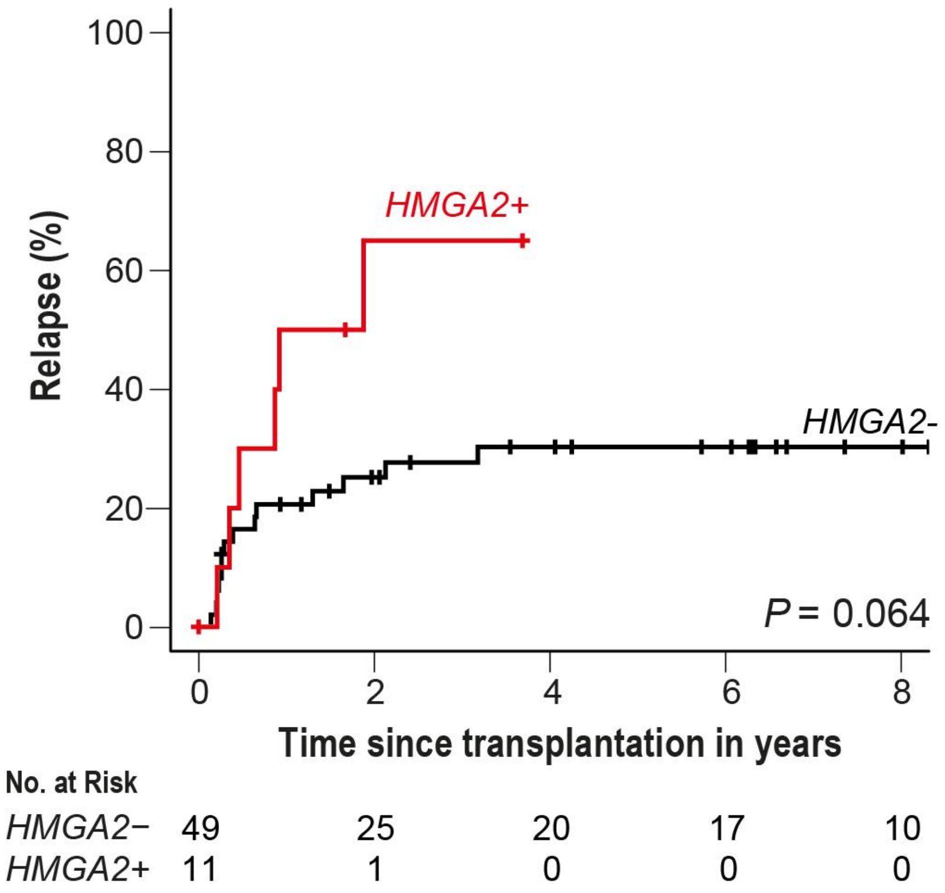

Figure S9. The 2017 ELN genetic risk stratification and the HMGA2 test.

Kaplan-Meier curves for overall survival (OS) of AML patients of the training cohort classified according to the 2017 ELN genetic risk stratification (left panel) and with the HMGA2 test (right panel). *H*+, high expression of *HMGA2* (≥1100 NCN); *H*-, low expression of *HMGA2* (<1100 NCN). The P values were obtained by the log-rank test for comparison of OS curves.

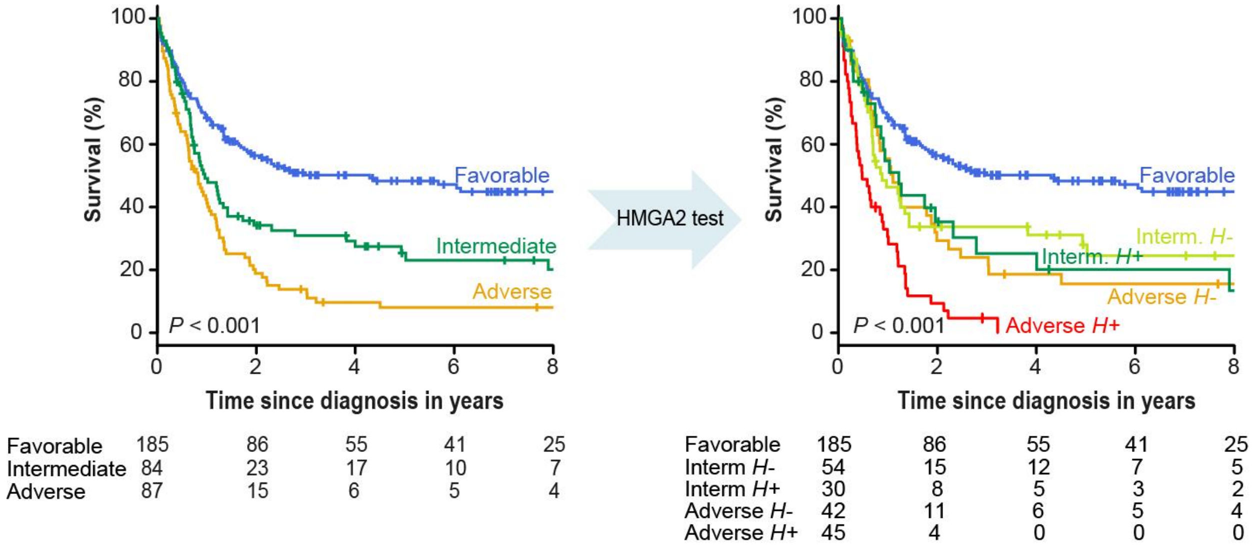

Figure S10. Proposed algorithm for laboratory testing in AML prognostic assessment.

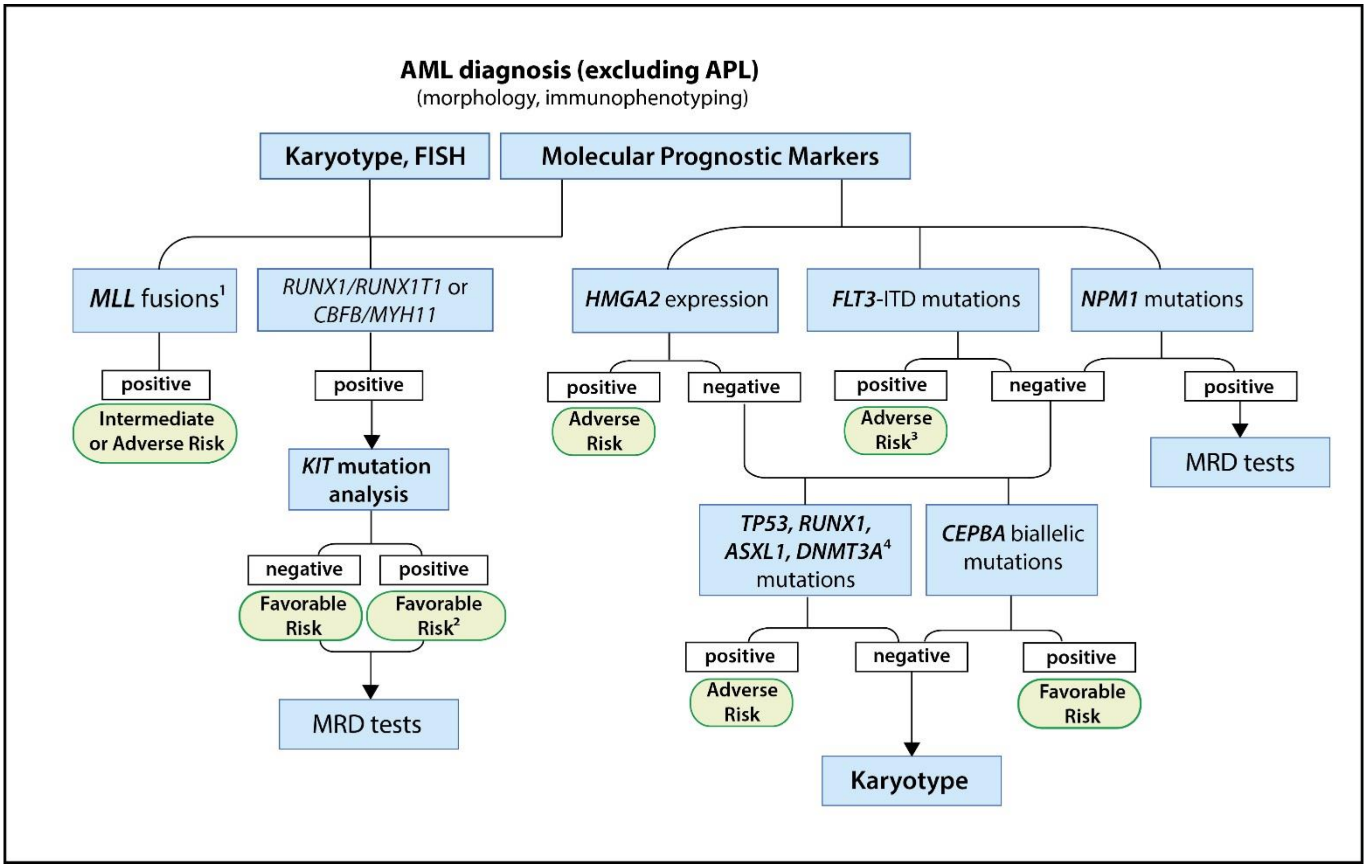

[1] The HMGA2 test is not informative in AML with *MLL* fusions.

[2] The presence of *KIT* mutations is associated with a higher risk of relapse mostly in AML with t(8;21). These patients are considered in the intermediate risk category in the NCCN guidelines for AML (Version 1.2018) but remain in the favorable genetic risk category in the 2017 ELN AML recommendations (Blood. 2017;129(4):424-447). MRD monitoring is recommended for these patients.

[3] The presence of *FLT3*-ITD mutations with a normal karyotype is considered in the poor risk category in the NCCN guidelines for AML (Version 1.2018). In the 2017 ELN AML recommendations, patients with wild-type *NPM1* and *FLT3*-ITD^{high} are classified in the adverse genetic risk category, patients with mutated *NPM1* and *FLT3*-ITD^{high} are classified in the intermediate genetic risk category as well as patients with wild-type *NPM1* without *FLT3*-ITD or with *FLT3*-ITD^{low}. Patients with mutated *NPM1* without *FLT3*-ITD or with *FLT3*-ITD^{low} are classified in the favorable genetic risk category.

[4] *RUNX1* and *ASXL1* mutations should be considered in the poor risk category only if they are not associated with favorable risk AML. The presence of *DNMT3A* mutations in combination with *NPM1* and *FLT3*-ITD mutations confers a poor prognosis in the study of Papaemmanuil E. *et al* (N Engl J Med 2016; 374:2209-21).

APL, acute promyelocytic leukemia; FISH, fluorescent *in situ* hybridization; *FLT3*-ITD^{high}, allelic ratio of *FLT3*-ITD ≥0.5, *FLT3*-ITD^{low}, allelic ratio of *FLT3*-ITD <0.5; MRD, minimal residual disease.

TABLES

Table S1. Clinical characteristics of *de novo* AML patients in the training cohort (n = 358)

Factor	Group	Overall
**Cohorts, n (%)**	BCLQ	95 (26.5)
	Leucegene	263 (73.5)
**Age at diagnosis, median (range)**		54 (17-78)
**Age at diagnosis, n (%)**	17-59 years	235 (65.6)
	≥60 years	123 (34.4)
**Gender, n (%)**	Female	164 (45.8)
	Male	194 (54.2)
**WBC count, n (%)**	<50 x 10^9/l	230 (65.0)
	50-99 x 10^9/l	76 (21.5)
	>100 x 10^9/l	48 (13.6)
**CR, n (%)**		279 (77.9)
**HSCT, n (%)**	HSCT CR1*	66 (18.4)
	HSCT CR2	32 (8.9)
	HSCT RD	2 (0.6)
**Cytogenetic risk, n (%)**	Favorable	54 (15.1)
	Intermediate	232 (64.8)
	Adverse	68 (19.0)
	Undetermined	4 (1.1)
***FLT3*-ITD†, n (%)**	Positive	98 (27.4)
	Negative	235 (65.6)
	Not available	25 (7.0)
***NPM1*‡, n (%)**	Positive	131 (36.6)
	Negative	202 (56.4)
	Not available	25 (7.0)
**biallelic *CEBPA* §, n (%)**	Positive	10 (12.7)
	Negative	69 (87.3)
**Median follow-up, y**		6.0
**Causes of death, n (%)**	Leukemia-related	141 (61.6)
	Other causes	46 (20.1)
	Not available	42 (18.3)

BCLQ, Banque de cellules leucémiques du Québec; CR, complete remission; HSCT, patients who received an allogeneic hematopoietic stem cell transplantation in first complete remission (CR1), in second complete remission (CR2) or in refractory disease (RD); ITD, internal tandem duplication; WBC, white blood cells.

*Number of patients who received an allogeneic hematopoietic stem cell transplantation in first complete remission in the different cytogenetic risk groups: favorable n = 2, adverse n = 18, intermediate n = 45, undetermined n = 1; 60 patients: <60 years old, 6 patients: ≥60 years old.

† *FLT3*-ITD mutations were evaluated in the clinical laboratory, n = 333 (allelic ratio ≥0.05 were considered positive)[4] or by RNA-sequencing, n = 25 (allelic ratio ≥0.1 were considered positive).[18]

‡*NPM1* and biallelic *CEPBA* mutations were tested in the clinical laboratory.

§Only patients negative for *FLT3*-ITD and *NPM1* mutations in the intermediate cytogenetic group (n = 79) were tested for biallelic *CEBPA* mutations.[19]

Table S2. Treatment regimens in the training cohort (n = 358)

TREATMENTS	n (%)
**Induction regimens (cycle 1)**	**358**
7+3 protocol (cytarabine + anthracyclin)	309 (86.3)
Anthracyclin / Cytarabine / Etoposide	20 (5.7)
7+3 protocol + experimental drug or placebo*	17 (4.7)
High-dose cytarabine + anthracyclin protocol	8 (2.2)
Other induction regimens†	4 (1.1)
**Consolidation regimens (cycle 1)**	**239**
High-dose cytarabine alone	222 (92.9)
High-dose cytarabine + anthracyclin (idarubicine, daunorubicine or mitoxantrone)	4 (1.7)
High-dose cytarabine + etoposide	5 (2.1)
Mitoxantrone + etoposide	1 (0.4)
Other consolidation regimens‡	7 (2.9)
**HSCT**	**104**
Autologous transplantation	4 (3.8)
HSCT in CR1	66 (63.5)
HSCT in CR2	32 (30.8)
HSCT in RD	2 (1.9)

All patients (n = 358) received from 1 to 3 cycles of induction therapy (1 cycle: 69% of patients, 2 cycles: 24.9%, 3 cycles: 6.1%); 239 patients in first complete remission received from 1 to 4 cycles of consolidation chemotherapy.

HSCT, patients who received an allogeneic hematopoietic stem cell transplantation in first complete remission (CR1), in second complete remission (CR2) or in refractory disease (RD).

* Experimental protocols: NCT00085709, n = 14; NCT00651261, n = 1; NCT01802333, n = 2.

†Other induction regimens: FLAG, HDAC/VP-16, cytarabine/etoposide, Mitoxantrone/VP-16 protocols, n = 1 each.

‡Other consolidation regimens: CALGB 8923, n = 3; NCT01802333, n = 2; NCT00317642, n = 2 (patients were refractory to the 7+3 induction regimen (1 cycle) and achieved a complete remission with induction regimen of the NCT00317642 protocol).

Table S3. Frequencies according to cytogenetic subgroups in the training cohort (n = 358)

**Cytogenetic subgroups**	**n (%)**
Normal karyotype	166 (46.4)
Intermediate abnormal karyotype	52 (14.5)
Complex karyotype*	36 (10.0)
inv(16)(p13.1q22)/t(16;16)(p13.1;q22); *CBFB-MYH11*	36 (10.0)
KMT2A (*MLL*) fusions	26 (7.3)
t(8;21)(q22;q22.1); *RUNX1-RUNX1T1*	18 (5.0)
Monosomy 5 or 5q-; Monosomy 7 or 7q- (not complex)	7 (2.0)
MECOM (*EVI1*) rearrangements	5 (1.4)
NUP98-NSD1 (normal karyotype)	5 (1.4)
Undetermined	4 (1.1)
t(6;9)(p23;q34); *DEK-NUP214*	2 (0.6)
t(9;22)(q34;q11.2); *BCR-ABL1*	1 (0.3)
**TOTAL**	358 (100.0)

*Includes 27 patients with monosomal and complex karyotypes.

Table S4. Frequencies according to the WHO 2008 categories in the training cohort (n = 358)

**WHO 2008 Classification**	**n (%)**
AML with t(8;21)(q22;q22.1); *RUNX1-RUNX1T1*	18 (5.0)
AML with inv(16)(p13.1q22) or t(16;16)(p13.1;q22); *CBFB-MYH11*	36 (10.1)
AML with t(9;11)(p22;q23); *MLLT3-MLL*	7 (2.0)
AML with t(6;9)(p23;q34); *DEK-NUP214*	2 (0.6)
AML with inv(3)(q21q26.2) or t(3;3)(q21;q26.2); *RPN1-EVI1*	2 (0.6)
AML with myelodysplasia-related changes	80 (22.3)
Acute myeloid leukaemia, NOS	18 (5.0)
AML with minimal differentiation	9 (2.5)
AML without maturation	83 (23.2)
AML with maturation	23 (6.4)
Acute myelomonocytic leukaemia	35 (9.8)
Acute monoblastic and monocytic leukaemia	38 (10.6)
Acute erythroid leukaemia	6 (1.7)
Acute megakaryoblastic leukaemia	1 (0.3)
**TOTAL**	358 (100.0)

Therapy-related AML, secondary AML and acute promyelocytic leukemia specimens were excluded from this study.

Table S5. Clinical characteristics of the Australian cohort (n = 70)

Factor	Group	Overall
**Age at diagnosis, median (range)**		48 (18-60)
**Age at diagnosis, n (%)**	18-59 years	68 (97.1)
	≥60 years	2 (2.9)
**Gender, n (%)**	Female	28 (40.0)
	Male	42 (60.0)
**WBC count, n (%)**	<50 x 10^9/l	47 (67.1)
	50-99 x 10^9/l	11 (15.7)
	>100 x 10^9/l	11 (15.7)
	Not available	1 (1.4)
**WHO 2016 classification, n (%)**	AML with t(8;21)(q22;q22.1); *RUNX1-RUNX1T1*	2 (2.9)
	AML with inv(16)(p13.1q22) or t(16;16)(p13.1;q22); *CBFB-MYH11*	8 (11.4)
	AML with t(9;11)(p22;q23); *MLLT3-KMT2A*	1 (1.4)
	AML with inv(3)(q21q26.2) or t(3;3)(q21;q26.2); *GATA2, MECOM*	2 (2.9)
	AML with mutated *NPM1*	12 (17.1)
	AML with biallelic mutations of *CEBPA*	2 (2.9)
	AML with myelodysplasia-related changes	18 (25.7)
	Therapy-related myeloid neoplasms	2 (2.9)
	AML with minimal differentiation	1 (1.4)
	AML without maturation	6 (8.6)
	AML with maturation	1 (1.4)
	Acute myelomonocytic leukaemia	6 (8.6)
	Acute monoblastic and monocytic leukaemia	6 (8.6)
	Acute erythroid leukaemia	1 (1.4)
	AML, transformed from myeloproliferative neoplasms	1 (1.4)
	MDS with excess blasts (RAEB2)	1 (1.4)
**HSCT, n (%)**	HSCT CR1	21 (30.0)
	HSCT CR2	10 (14.3)
**Cytogenetic risk*, n (%)**	Favorable	10 (14.3)
	Intermediate	40 (57.1)
	Adverse	15 (21.4)
	Unknown	5 (7.1)
***FLT3*-ITD, n (%)**	Positive	16 (24.2)
	Negative	50 (75.7)
	Not available	4 (5.7)
**CR*, n (%)**		59 (84.3)
**Median follow-up, y**		2.8

CR, complete remission; HSCT, patients who received an allogeneic hematopoietic stem cell transplantation in first complete remission (CR1) or in second complete remission (CR2); ITD, internal tandem duplication; MDS, myelodysplastic syndrome; RAEB2, Refractory Anaemia with Excess Blasts type 2; WBC, white blood cells.

* In this cohort, 17 of 25 *HMGA2*+ patients obtained a CR compared to 42 of 45 *HMGA2*- patients (68.0% vs 93.3%, *P* =0.008).

Table S6. Primers and probe used for the HMGA2 RT-qPCR test

Gene		Primers and probe sequences
	Forward primer	CACTTCAGCCCAGGGACAA
HMGA2	Reverse primer	CTCACCGGTTGGTTCTTGCT
	Probe	CTCAGAAGAGAGGACGCGGCC

Table S7. Analytical validation of the HMGA2 RT-qPCR test

Parameter		HMGA2 test performance
Specificity (%)		100
PCR Efficiency (%) (mean ± SD)		100.55 ± 1.63
Linearity R^2 (mean ± SD)		0.999 ± 0.002
Analytical sensitivity (LoQ; copies/5 µl)		100
Reproducibility	Precision – Repeatability (within-run) copies/5 µl, %CV (CI)	6.71 (2.59 – 16.0)
	Intermediate Precision (day-to-day) copies/5 µl, %CV (CI)	8.61 (4.84 – 12.38)
	Robustness – MasterMix lot-to-lot, copies/5 µl, %CV (CI)	8.71 (6.01 – 11.41)
	Robustness – system-to-system, copies/5 µl, %CV (CI)	8.65 (5.97 – 11.32)
Reportable Range (copies/5 µl)		10^2 to 10^6

CI, confidence interval; CV, coefficient of variation; LoQ, limit of quantification; PCR, polymerase chain reaction; SD, standard deviation.

Table S8. Compliance with REMARK guidelines

REMARK checklist	Information in this paper
**INTRODUCTION**	
1. State the marker examined, the study objectives, and any pre-specified hypotheses.	✓ Introduction and results (main paper)
**MATERIALS AND METHODS**	
Patients	
2. Describe the characteristics of the study patients, including their source and inclusion and exclusion criteria.	✓ Characteristics of the studied patients (training cohort): Figure 1, Supplementary Table S1, Supplementary Figures S1, S2<br>✓ Cytogenetic subgroups: Supplementary Table S3<br>✓ WHO 2008 categories: Supplementary Table S4<br>✓ Source and inclusion/exclusion criteria: Methods (main paper)<br>✓ External validation cohort: Table 1
3. Describe treatments received and how chosen.	✓ Treatments (training cohort): Supplementary Table S2
Specimen characteristics	
4. Describe type of biological material used (including control samples) and methods of preservation and storage.	✓ Methods (main paper and Supplementary Information)
Assay methods	
5. Specify the assay method used and provide (or reference) a detailed protocol, including specific reagents or kits used, quality control procedures, reproducibility assessments, quantitation methods, and scoring and reporting protocols. Specify whether and how assays were performed blinded to the study endpoint.	✓ Methods (main paper and Supplementary Information)
Study design	
6. State the method of case selection, including whether prospective or retrospective and whether stratification or matching (for example, by stage of disease or age) was used. Specify the time period from which cases were taken, the end of the follow-up period, and the median follow-up time.	✓ Methods (main paper)
7. Precisely define all clinical endpoints examined.	✓ Methods (main paper)
8. List all candidate variables initially examined or considered for inclusion in models.	✓ Methods (main paper)
9. Give rationale for sample size; if the study was designed to detect a specified effect size, give the target power and effect size.	✓ Statistical methods (Supplementary Information)
Statistical analysis methods	
10. Specify all statistical methods, including details of any variable selection procedures and other model-building issues, how model assumptions were verified, and how missing data were handled.	✓ Statistical methods (main paper)<br>✓ PH assumption verification: Statistical methods (Supplementary Information)<br>✓ Handling of missing data: Statistical methods (Supplementary Information)
11. Clarify how marker values were handled in the analyses; if relevant, describe methods used for cutpoint determination.	✓ Methods (main paper)

RESULTS	
Data	
12. Describe the flow of patients through the study, including the number of patients included in each stage of the analysis and reasons for dropout. Specifically, both overall and for each subgroup extensively examined report the number of patients and the number of events.	✓ Not applicable
13. Report distributions of basic demographic characteristics (at least age and sex), standard (disease-specific) prognostic variables, and tumor marker, including numbers of missing values.	✓ Characteristics of patients: Supplementary Table S1<br>✓ Cytogenetic subgroups: Supplementary Table S3
Analysis and presentation	
14. Show the relation of the marker to standard prognostic variables.	✓ Table 1 and Figure 2 (main paper)
15. Present univariate analyses showing the relation between the marker and outcome, with the estimated effect. Preferably provide similar analyses for all other variables being analyzed. For the effect of a tumor marker on a time-to-event outcome, a Kaplan-Meier plot is recommended.	✓ Univariate analyses: Supplementary Table S11<br>✓ Kaplan-Meier plot and cumulative incidence of relapse curves: Figure 3 and Figure 5 (main paper), Supplementary Figures S8 and Supplementary Table S10
16. For key multivariable analyses, report estimated effects with confidence intervals for the marker and, at least for the final model, all other variables in the model.	✓ Figure 4 (main paper), Table 2 and Supplementary Table S12
17. Among reported results, provide estimated effects with confidence intervals from an analysis in which the marker and standard prognostic variables are included, regardless of their statistical significance.	✓ Figure 4 (main paper), Table 2 and Supplementary Table S12
18. If done, report results of further investigations, such as checking assumptions, sensitivity analyses, and internal validation.	✓ PH assumption verification: Statistical methods (Supplementary Information)<br>✓ External validation of the HMGA2 test in the NCRI AML17 cohort: main text, Figure 3, Table 3
**DISCUSSION**	
19. Interpret the results in the context of the pre-specified hypotheses and other relevant studies; include a discussion of limitations of the study.	✓ Discussion (main paper)
20. Discuss implications for future research and clinical value.	✓ Discussion (main paper)

Table S9. Results of multivariable analyses for *PAWR* in the NCRI AML17 validation cohort

Outcome	*PAWR-*	*PAWR+*	Unadjusted OR/HR (95% CI) *P*	Adjusted* OR/HR (95% CI) *P*
	n = 134	n = 129		
**CR/CRi****	93%	86%	2.19 (0.99-4.84)<br>0.05	1.96 (0.75-5.10)<br>0.17
**Overall survival**	46%†	33%†	1.16 (0.83-1.64)<br>0.4	1.01 (0.69-1.47)<br>1.0
**Relapse-free survival**	44%†	27%†	1.43 (1.02-2.11)<br>0.04	1.47 (1.01-2.14)<br>0.05
**Cumulative incidence of relapse**	46%†	57%†	1.37 (0.93-2.01)<br>0.11	1.46 (0.96-2.21)<br>0.07

CI, confidence intervals; HR, hazard ratio; OR, odds ratio; *PAWR-,* low expression level; *PAWR+,* high expression level.
*Variables included in the multivariable models are: age, log white blood cell count, secondary disease, WHO/ECOG performance status, the presence of adverse cytogenetics, *FLT3*-ITD and *NPM1* mutations. **Complete remission (CR) and Complete remission with incomplete hematologic recovery (CRi) excluding induction deaths. †Clinical end-points at 5 years.

Note: The cut-off for the PAWR test was determined following the same methodology as for the HMGA2 test.

Table S10. Overall survival, relapse-free survival and cumulative incidence of relapse frequencies at 3 years in the training cohort, the intermediate cytogenetic risk patients and in transplanted patients under 60 years old

The cut-off of the test was established at 1100 NCN. *HMGA2*+, high expression (≥1100 NCN); *HMGA2*-, low expression (<1100 NCN).

		Overall survival			Relapse-free survival			Cumulative incidence of relapse		
**Groups**	***HMGA2* status**	**n**	**survival rate % (95% CI)**	***P***	**n**	**survival rate % (95% CI)**	***P***	**n**	**incidence of relapse % (95% CI)**	***P***
**Training cohort**	–	278	43.5 (37.3 – 49.5)	6.3e-08	232	44.2 (37.4 – 50.7)	7.1e-05	232	48.1 (41.2 – 54.6)	4.0e-03
	+	80	13.2 (6.5 – 22.4)		47	10.8 (3.2 – 23.7)		47	72.9 (54.6 – 84.8)	
**Intermediate cytogenetic risk (training cohort)**	-	191	40.2 (32.8 – 47.4)	0.024	157	42.0 (33.8 – 49.9)	0.010	157	50.6 (42.2 – 58.5)	0.028
	+	41	17.3 (6.8 – 31.8)		28	12.8 (2.8 – 30.8)		28	75.6 (48.8 – 89.7)	
**HSCT in CR1 patients under 60 years old**	–	49	63.6 (48.0 – 75.7)	0.013	49	57.6 (42.1 – 70.3)	0.047	49	27.7 (15.6 – 41.1)	0.064
	+	11	13.3 (0.8 – 43.3)		11	15.0 (1.0 – 45.7)		11	65.0 (17.0 – 90.0)	

CI, confidence interval; CR1, first complete remission; HSCT, allogeneic hematopoietic stem cell transplantation.

Table S11. Univariate analysis of complete remission, relapse-free survival, overall survival, and cumulative incidence of relapse in the training cohort

Outcome	Variables	OR or HR (95% CI)	*P*
Complete Remission	Age at diagnosis	1.04 (1.01-1.06)	0.001
	WBC ≥100 *vs* WBC <100	1.27 (0.62-2.59)	0.510
	NPM1+ vs NPM1-	0.82 (0.47-1.41)	0.471
	FLT3-ITD+ *vs FLT3*-ITD-	1.29 (0.74-2.26)	0.375
	Adverse *vs* favorable cytogenetic risk	8.70 (2.39-31.70)	0.001
	Intermediate *vs* favorable cytogenetic risk	3.44 (1.02-11.60)	0.047
	HMGA2+ vs HMGA2-	3.62 (2.00-6.54)	<0.001
Relapse-Free Survival	Age at diagnosis	1.03 (1.02-1.05)	<0.001
	WBC ≥100 *vs* WBC <100	0.97 (0.61-1.55)	0.898
	NPM1+ vs NPM1-	1.21 (0.88-1.66)	0.230
	FLT3-ITD+ vs *FLT3*-ITD-	1.50 (1.07-2.10)	0.018
	Adverse *vs* favorable cytogenetic risk	4.50 (2.51-8.06)	<0.001
	Intermediate *vs* favorable cytogenetic risk	2.72 (1.63-4.52)	<0.001
	HSCT	0.90 (0.62-1.33)	0.609
	HMGA2+ vs HMGA2-	1.99 (1.37-2.88)	0.001
Overall Survival	Age at diagnosis	1.04 (1.03-1.05)	<0.001
	WBC ≥100 *vs* WBC <100	1.08 (0.74-1.59)	0.693
	NPM1+ vs NPM1-	1.12 (0.85-1.48)	0.433
	FLT3-ITD+ *vs FLT3*-ITD-	1.56 (1.71-2.09)	0.002
	Adverse *vs* favorable cytogenetic risk	6.10 (3.49-10.63)	<0.001
	Intermediate *vs* favorable cytogenetic risk	3.10 (1.84-5.19)	<0.001
	HSCT	0.78 (0.53-1.15)	0.207
	HMGA2+ vs HMGA2-	2.15 (1.61-2.87)	<0.001
Cumulative Incidence of relapse	Age at diagnosis	1.04 (1.02 - 1.05)	<0.001
	WBC ≥100 *vs* WBC <100	1.04 (0.63 - 1.70)	0.888
	NPM1+ vs NPM1-	1.21 (0.86 - 1.70)	0.281
	FLT3-ITD+ *vs FLT3*-ITD-	1.46 (1.02 - 2.10)	0.042
	Adverse vs favorable cytogenetic risk	3.91 (2.07 – 7.37)	<0.001
	Intermediate vs favorable cytogenetic risk	2.65 (1.54 – 4.56)	<0.001
	HSCT	0.60 (0.39 - 0.95)	0.028
	HMGA2+ vs HMGA2-	1.85 (1.23 - 2.80)	0.003

CI, confidence intervals; *HMGA2+*, high expression (≥1100 NCN); *HMGA2-*, low expression (<1100 NCN); HR, hazard ratio; HSCT, allogeneic hematopoietic stem cell transplantation; ITD, internal tandem duplication; OR, odds ratio; vs, versus; WBC, white blood cells (x 10^9/l).

Table S12. Results of multivariable analysis for overall survival adjusted for the ELN poor risk mutations *TP53, ASXL1* and *RUNX1* in the sequenced cohort (n = 263)

Variables	aHR (95% CI)	*P*
WBC ≥100 vs WBC <100	1.14 (0.76-1.72)	0.520
NPM1	0.64 (0.39-1.06)	0.082
FLT3-ITD	0.89 (0.48-1.63)	0.697
NPM1 / *FLT3*-ITD interaction	3.9 (1.78-8.53)	0.001
Adverse vs favorable cytogenetic risk	6.47 (3.21-13.04)	<0.001
Intermediate vs favorable cytogenetic risk	3.00 (1.54-5.83)	0.001
HSCT	0.53 (0.32-0.87)	0.012
HMGA2+ vs *HMGA2*-	1.73 (1.06-2.84)	0.030
ASXL1	1.06 (0.5-2.26)	0.876
RUNX1	1.02 (0.55-1.87)	0.958
TP53 *	1.57 (0.8-3.1)	0.190

Since the non-linear effect of age at diagnosis is represented jointly by the 2 coefficients (linear and quadratic), the interpretation of each coefficient separately is not appropriate. See statistical methods (Supplementary Information) for description of the adjusted effect of age at diagnosis.
aHR, adjusted hazard ratio; CI, confidence intervals; *HMGA2*+, high expression (≥1100 NCN); *HMGA2*-, low expression (<1100 NCN); ITD, internal tandem duplication; WBC, white blood cells (x 10^9/l).
*The variable *TP53* mutation is significant (aHR=2.23, (95% CI, 1.22-4.08), *P* = 0.009) if *HMGA2* is not included in the multivariable model for overall survival.

Table S13. Multivariable analysis of complete remission, relapse-free survival, overall survival, and cumulative incidence of relapse in sequenced patients of the training cohort classified in the intermediate cytogenetic risk category (n = 163)

Outcome	Variables	aOR or aHR (95% CI)	*P*
Complete Remission	WBC ≥100 *vs* WBC <100	1.47 (0.56-3.86)	0.432
	NPM1	0.58 (0.15-2.30)	0.441
	FLT3-ITD	0.85 (0.18-4.00)	0.839
	NPM1 / FLT3-ITD interaction	4.77 (0.68-33.5)	0.116
	RUNX1	1.70 (0.43-6.71)	0.452
	ASXL1	0.95 (0.17-5.22)	0.952
	HMGA2+ vs HMGA2-	3.27 (0.95-11.30)	0.061
Relapse-Free Survival	WBC ≥100 *vs* WBC <100	1.45 (0.79-2.68)	0.233
	NPM1	0.62 (0.34-1.15)	0.133
	FLT3-ITD	0.59 (0.22-1.62)	0.311
	NPM1 / FLT3-ITD interaction	4.18 (1.31-13.33)	0.016
	HSCT	0.48 (0.24-0.94)	0.032
	RUNX1	1.26 (0.49-3.23)	0.631
	ASXL1	0.74 (0.24-2.33)	0.61
	biallelic *CEBPA*	0.26 (0.07-0.92)	0.036
	HMGA2+ vs HMGA2-	2.67 (1.24-5.77)	0.012
Overall Survival	WBC ≥100 *vs* WBC <100	1.48 (0.90-2.43)	0.121
	NPM1	0.60 (0.34-1.06)	0.076
	FLT3-ITD	1.04 (0.47-2.32)	0.918
	NPM1 / FLT3-ITD interaction	3.12 (1.22-7.99)	0.018
	HSCT	0.43 (0.22-0.85)	0.015
	RUNX1	0.82 (0.39-1.70)	0.592
	ASXL1	1.22 (0.53-2.83)	0.635
	biallelic *CEBPA*	0.29 (0.09-1.00)	0.05
	HMGA2+ vs HMGA2-	2.38 (1.26-4.50)	0.008
Cumulative incidence of relapse	WBC ≥100 *vs* WBC <100	1.51 (0.79-2.90)	0.214
	NPM1	0.58 (0.30-1.11)	0.102
	FLT3-ITD	0.43 (0.13-1.40)	0.16
	NPM1 / FLT3-ITD interaction	6.20 (1.61-23.81)	0.008
	HSCT	0.43 (0.20-0.91)	0.027
	RUNX1	1.44 (0.54-3.86)	0.467
	ASXL1	0.58 (0.17-2.00)	0.392
	biallelic *CEBPA*	0.20 (0.04-0.90)	0.036
	HMGA2+ vs HMGA2-	2.61 (1.13-6.05)	0.025

aHR, adjusted hazard ratio; aOR, adjusted odds ratio; CI, confidence intervals; *HMGA2+*, high expression (≥1100 NCN); *HMGA2-*, low expression (<1100 NCN); HSCT, allogeneic hematopoietic stem cell transplantation; ITD, internal tandem duplication; vs, versus; WBC, white blood cells (x 10^9/l).

Note: Only two of the 165 sequenced intermediate cytogenetic risk patients had *TP53* mutations and were excluded from these analyses.

Biallelic *CEBPA* mutational status was not included in the complete remission model because all bi*CEBPA* mutated patients achieved a complete remission.

Since the non-linear effect of age at diagnosis is represented jointly by the 2 coefficients (linear and quadratic), the interpretation of each coefficient separately is not appropriate and is not represented in the models.

Table S14. 2017 ELN genetic risk stratification and *HMGA2* expression levels in the training cohort (n = 358)

The cut-off of the HMGA2 test was established at 1100 NCN*.* *HMGA2*-, low expression (<1100 NCN); *HMGA2+*, high expression (≥1100 NCN).

	Number of patients		
	HMGA2-	***HMGA2+***	**Total**
**Favorable risk**	**180**	**5**	**185**
t(8;21)(q22;q22.1); *RUNX1-RUNX1T1*	17	1	18
inv(16)(p13.1q22) or t(16;16)(p13.1;q22); *CBFB-MYH11*	34	2	36
Biallelic mutated *CEBPA*	12	0	12
Mutated *NPM1* with *FLT3*-ITD^{low*}	54	0	54
Mutated *NPM1* without *FLT3*-ITD	63	2	65
**Intermediate risk**	**54**	**30**	**84**
Mutated *NPM1* with *FLT3*-ITD^{high*}	10	0	10
t(9;11)(p21.3;q23.3); *MLLT3-KMT2A*	7	0	7
Wild type *NPM1* without *FLT3*-ITD or with *FLT3*-ITD^{low*} (without adverse-risk genetic lesions)	23	10	33
Cytogenetic abnormalities not classified as favorable or adverse	14	20	34
**Adverse risk**	**42**	**45**	**87**
t(6;9)(p23;q34.1); *DEK-NUP214*	2	0	2
t(v;11q23.3); *KMT2A* rearranged (excluding t(9;11))	16	2	18
t(9;22)(q34.1;q11.2); *BCR-ABL1*	1	0	1
inv(3)(q21.3q26.2) or t(3;3)(q21.3;q26.2); *GATA2,MECOM(EVI1*)	2	3	5
-5 or del(5q); -7 or del(7q) ; -17/abn(17p)	6	1	7
Complex karyotype without adverse genetic lesions	3	3	6
Monosomal karyotype (wild-type *TP53* or not done)	2	12	14
Monosomal karyotype and mutated *TP53*	1	12	13
Wild-type *NPM1* with *FLT3*-ITD^{high}	2	1	3
Mutated *RUNX1* only†	3	4	7
Mutated *ASXL1* only†	2	1	3
Mutated *RUNX1* and *ASXL1*†	2	3	5
Mutated *TP53*‡	0	3	3
**Undetermined**	**2**	**0**	**2**

**FLT3*-ITD mutations were evaluated in the clinical laboratory; low, low allelic ratio (<0.5); high, high allelic ratio (≥0.5).

†According to the 2017 ELN AML recommendations, these mutations were not classified as adverse risk if they co-occur with favorable risk AML subtypes. All these patients had intermediate risk cytogenetics.

‡One patient also had a *RUNX1* mutation. Two patients had a complex karyotype and 1 had a hyperdiploid karyotype (numerical abnormalities only).

Seven patients in the adverse risk *H+* group received an allogeneic stem cell transplantation in first complete remission.

REFERENCES

1. Falini B, Mecucci C, Tiacci E, Alcalay M, Rosati R, Pasqualucci L, *et al.* Cytoplasmic nucleophosmin in acute myelogenous leukemia with a normal karyotype. *The New England journal of medicine* 2005 Jan 20; **352**(3)**:** 254-266.

2. Pabst T, Mueller BU, Zhang P, Radomska HS, Narravula S, Schnittger S, *et al.* Dominant-negative mutations of CEBPA, encoding CCAAT/enhancer binding protein-alpha (C/EBPalpha), in acute myeloid leukemia. *Nature genetics* 2001 Mar; **27**(3)**:** 263-270.

3. Gale RE, Green C, Allen C, Mead AJ, Burnett AK, Hills RK, *et al.* The impact of FLT3 internal tandem duplication mutant level, number, size, and interaction with NPM1 mutations in a large cohort of young adult patients with acute myeloid leukemia. *Blood* 2008 Mar 1; **111**(5)**:** 2776-2784.

4. Linch DC, Hills RK, Burnett AK, Khwaja A, Gale RE. Impact of FLT3(ITD) mutant allele level on relapse risk in intermediate-risk acute myeloid leukemia. *Blood* 2014 Jul 10; **124**(2)**:** 273-276.

5. Untergasser A, Cutcutache I, Koressaar T, Ye J, Faircloth BC, Remm M, *et al.* Primer3--new capabilities and interfaces. *Nucleic acids research* 2012 Aug; **40**(15)**:** e115.

6. Gabert J, Beillard E, van der Velden VH, Bi W, Grimwade D, Pallisgaard N, *et al.* Standardization and quality control studies of 'real-time' quantitative reverse transcriptase polymerase chain reaction of fusion gene transcripts for residual disease detection in leukemia - a Europe Against Cancer program. *Leukemia* 2003 Dec; **17**(12)**:** 2318-2357.

7. Sabath DE, Algar E, Bhattacharyya PK, Bijwaard KE, Hong T, Lindeman N, *et al.* Nucleic acid amplification assays for molecular hematopathology; approved guideline. *2nd edition Wayne PA: NCCLS; CLSI document MM05-A2* 2012.

8. Budd JR, Durham AP, Gwise TE, Iriarte B, Kallner A, Linnet K, *et al.* Measurement procedure comparison and bias estimation using patient samples; approved guideline. . *3rd edition Wayne PA: NCCLS; CLSI document EP09-A3* 2013.

9. Horowitz GL, Altaie S, Boyd JC, Ceriotti F, Garg U, Horn P*, et al.* Defining, establishing, and verifying reference intervals in the clinical laboratory; approved guideline. *3rd edition Wayne PA: NCCLS; CLSI document EP28-A3* 2010.

10. McEnroe RJ, Durham AP, Goldford MD, Kondratovich MV, Lababidi S, Magari R*, et al.* Evaluation of precision of quantitative measurement procedure; approved guideline. *3rd edition Wayne PA: NCCLS; CLSI document EP05-A3* 2014.

11. Pierson-Perry JF, Vaks JE, Durham AP, Fischer C, Gutenbrunner C, Hiiyard D*, et al.* Evaluation of detection capability for clinical laboratory measurement procedures; approved guideline. 2nd edition. *Wayne PA: NCCLS; CLSI document EP17-A2* 2012.

12. Tholen DW, Kroll M, Astles JR, Caffo AL, Happe TM, Krouwer J*, et al.* Evaluation of the linearity of quantitative measurement procedures: a statistical approach; approved guideline. . *Wayne PA: NCCLS; CLSI document EP06-A* 2003.

13. Bustin SA, Benes V, Garson JA, Hellemans J, Huggett J, Kubista M*, et al.* The MIQE guidelines: minimum information for publication of quantitative real-time PCR experiments. *Clin Chem* 2009 Apr; **55**(4)**:** 611-622.

14. Burd EM. Validation of laboratory-developed molecular assays for infectious diseases. *Clinical microbiology reviews* 2010 Jul; **23**(3)**:** 550-576.

15. Benedetti A, Abrahamowicz M. Using generalized additive models to reduce residual confounding. *Statistics in medicine* 2004 Dec 30; **23**(24)**:** 3781-3801.

16. Burnett AK, Hills RK, Wheatley K, Goldstone AH, Prentice AG, Milligan D. A sensitive risk score for directing treatment in younger patients with AML. *Blood* 2006; **108**(11)**:** 18.

17. Ling V, Burnett AK, Bradstock K, Seymour JF, Hills RK, Wei A. Utility of a clinical risk score to identify high-risk patients with de novo acute myeloid leukaemia in first remission after high-dose cytarabine (HiDAC) based induction chemotherapy. *British journal of haematology* 2013 Mar; **160**(6)**:** 861-863.

18. Lavallee VP, Krosl J, Lemieux S, Boucher G, Gendron P, Pabst C*, et al.* Chemo-genomic interrogation of CEBPA mutated AML reveals recurrent CSF3R mutations and subgroup sensitivity to JAK inhibitors. *Blood* 2016 Jun 16; **127**(24)**:** 3054-3061.

19. Green CL, Koo KK, Hills RK, Burnett AK, Linch DC, Gale RE. Prognostic significance of CEBPA mutations in a large cohort of younger adult patients with acute myeloid leukemia: impact of double CEBPA mutations and the interaction with FLT3 and NPM1 mutations. *J Clin Oncol* 2010 Jun 1; **28**(16)**:** 2739-2747.
